# Characterization of group I introns in generating circular RNAs as vaccines

**DOI:** 10.1093/nar/gkaf089

**Published:** 2025-02-28

**Authors:** Kuo-Chieh Liao, Majid Eshaghi, Zebin Hong, Tzuen Yih Saw, Jian An Jovi Lim, Jian Han, Jong Ghut Ashley Aw, Kiat Yee Tan, Aixin Yap, Xiang Gao, Youzhi Anthony Cheng, Su Ying Lim, You Zhi Nicholas Cheang, Wilfried A A Saron, Abhay P S Rathore, Li Zhang, Bhuvaneshwari Shunmuganathan, Rashi Gupta, Siang Ling Isabelle Tan, Xinlei Qian, Kiren Purushotorman, Nagavidya Subramaniam, Leah A Vardy, Paul A Macary, Ashley John, Yi Yan Yang, Sylvie Alonso, Haiwei Song, Roland G Huber, Yue Wan

**Affiliations:** Genome Institute of Singapore, Agency for Science, Technology and Research (A*STAR), 138672, Singapore; Genome Institute of Singapore, Agency for Science, Technology and Research (A*STAR), 138672, Singapore; Institute of Molecular and Cell Biology, Agency for Science, Technology and Research (A*STAR), 138673, Singapore; Genome Institute of Singapore, Agency for Science, Technology and Research (A*STAR), 138672, Singapore; Genome Institute of Singapore, Agency for Science, Technology and Research (A*STAR), 138672, Singapore; Genome Institute of Singapore, Agency for Science, Technology and Research (A*STAR), 138672, Singapore; Genome Institute of Singapore, Agency for Science, Technology and Research (A*STAR), 138672, Singapore; Genome Institute of Singapore, Agency for Science, Technology and Research (A*STAR), 138672, Singapore; Genome Institute of Singapore, Agency for Science, Technology and Research (A*STAR), 138672, Singapore; Genome Institute of Singapore, Agency for Science, Technology and Research (A*STAR), 138672, Singapore; Genome Institute of Singapore, Agency for Science, Technology and Research (A*STAR), 138672, Singapore; Genome Institute of Singapore, Agency for Science, Technology and Research (A*STAR), 138672, Singapore; Infectious Diseases Translational Research Programme, Department of Microbiology & Immunology, Yong Loo Lin School of Medicine, National University of Singapore, 119077, Singapore; Immunology programme, Life Sciences Institute, National University of Singapore, 119077, Singapore; Program in Emerging Infectious Diseases, Duke-NUS Medical School, 169857, Singapore; Program in Emerging Infectious Diseases, Duke-NUS Medical School, 169857, Singapore; Pathology Department, Duke University Medical Center, Durham, NC 27708, United States; Bioprocessing Technology Institute, Agency for Science, Technology and Research (A*STAR), 138668, Singapore; Department of Microbiology & Immunology, Yong Loo Lin School of Medicine, National University of Singapore, 119077, Singapore; Antibody Engineering Programme, Life Sciences Institute, National University of Singapore, 119077, Singapore; Antibody Engineering Programme, Life Sciences Institute, National University of Singapore, 119077, Singapore; NUH-Cambridge Immune Phenotyping Centre, Life Sciences Institute, National University of Singapore, 119077, Singapore; NUH-Cambridge Immune Phenotyping Centre, Life Sciences Institute, National University of Singapore, 119077, Singapore; Department of Microbiology & Immunology, Yong Loo Lin School of Medicine, National University of Singapore, 119077, Singapore; Antibody Engineering Programme, Life Sciences Institute, National University of Singapore, 119077, Singapore; Department of Microbiology & Immunology, Yong Loo Lin School of Medicine, National University of Singapore, 119077, Singapore; Antibody Engineering Programme, Life Sciences Institute, National University of Singapore, 119077, Singapore; A*STAR Skin Research Labs and Skin Research Institute of Singapore, A*STAR, Immunos, 138648, Singapore; A*STAR Skin Research Labs and Skin Research Institute of Singapore, A*STAR, Immunos, 138648, Singapore; Department of Microbiology & Immunology, Yong Loo Lin School of Medicine, National University of Singapore, 119077, Singapore; Antibody Engineering Programme, Life Sciences Institute, National University of Singapore, 119077, Singapore; NUH-Cambridge Immune Phenotyping Centre, Life Sciences Institute, National University of Singapore, 119077, Singapore; Program in Emerging Infectious Diseases, Duke-NUS Medical School, 169857, Singapore; Pathology Department, Duke University Medical Center, Durham, NC 27708, United States; Department of Microbiology & Immunology, Yong Loo Lin School of Medicine, National University of Singapore, 119077, Singapore; Bioprocessing Technology Institute, Agency for Science, Technology and Research (A*STAR), 138668, Singapore; Infectious Diseases Translational Research Programme, Department of Microbiology & Immunology, Yong Loo Lin School of Medicine, National University of Singapore, 119077, Singapore; Immunology programme, Life Sciences Institute, National University of Singapore, 119077, Singapore; Institute of Molecular and Cell Biology, Agency for Science, Technology and Research (A*STAR), 138673, Singapore; Bioinformatics Institute, Agency for Science, Technology and Research (A*STAR), 138671, Singapore; Genome Institute of Singapore, Agency for Science, Technology and Research (A*STAR), 138672, Singapore

## Abstract

Circular RNAs are an increasingly important class of RNA molecules that can be engineered as RNA vaccines and therapeutics. Here, we screened eight different group I introns for their ability to circularize and delineated different features that are important for their function. First, we identified the *Scytalidium dimidiatum* group I intron as causing minimal innate immune activation inside cells, underscoring its potential to serve as an effective RNA vaccine without triggering unwanted reactogenicity. Additionally, mechanistic RNA structure analysis was used to identify the P9 domain as important for circularization, showing that swapping sequences can restore pairing to improve the circularization of poor circularizers. We also determined the diversity of sequence requirements for the exon 1 and exon 2 (E1 and E2) domains of different group I introns and engineered a S1 tag within the domains for positive purification of circular RNAs. In addition, this flexibility in E1 and E2 enables substitution with less immunostimulatory sequences to enhance protein production. Our work deepens the understanding of the properties of group I introns, expands the panel of introns that can be used, and improves the manufacturing process to generate circular RNAs for vaccines and therapeutics.

## Graphical Abstract

**Graphical Abstract:**
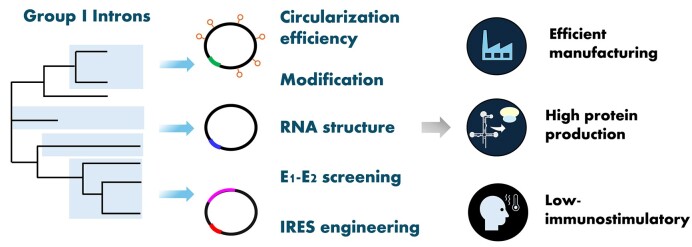

## Introduction

Nucleic acid vaccines based on messenger RNA (mRNA) technology hold great promise for rapid response towards infectious disease outbreaks and the recent success of the SARS-CoV-2 mRNA vaccine has saved tens of millions of lives globally [1]. These mRNA vaccines contain several structural components, including a 5′ cap, a 5′ untranslated region (UTR) sequence, an open reading frame that encodes an antigen of interest, a 3′ UTR sequence, and 3′ poly(A) tail [2]. Importantly, *N*^1^-methylpseudouridine is incorporated to reduce unwanted innate immune activation and to increase protein production inside cells [3]. More recently, circular RNA has attracted much attention as this RNA species does not have free ends and could serve as a more durable platform for protein expression in cells [4].

Circular RNAs were first identified in cells more than a decade ago [5, 6] and have been shown to be stable, with the ability to produce proteins inside cells [7]. Various groups have engineered circular RNAs to increase their translatability and stability inside cells for many applications, including RNA vaccines [8, 9]. A key aspect of these efforts is to maximize protein production potential from circular RNAs in a tuneable manner. This is mostly achieved by inserting optimized internal ribosome entry site (IRES) sequences and regulatory motifs into circular RNAs [10–12]. Unlike endogenous circular RNA, which is primarily produced through back-splicing in cells, several techniques are employed to produce circular RNA in test tubes [13–18]. Among these techniques, the utilization of auto-splicing group I intron ribozyme sequences in permuted intron–exon (PIE) systems appears to be an efficient strategy [8, 19–21].

Group I introns are catalytic RNA elements that can undergo catalytic reactions to self-splicing [19]. This reaction is initiated by a nucleophilic attack by the 3′ hydroxyl group of an exogenous GTP at the 5′ splice site. Subsequently, a second transesterification reaction occurs to excise the intron and ligate the exon. Although group I introns are extremely diverse and occur in many different organisms, most circular RNAs are generated using *T4 phage* and *Anabaena* introns [8, 21, 22]. However, it remains unclear how efficiently and accurately group I introns from other organisms work in circularization, what their structures look like, whether the circles that they generate can induce an innate immune response, and how they can be better engineered and manufactured. Here, we characterized eight different group I introns (*T4 phage* and *Anabaena* introns, as well as six others) in PIE systems to better understand their properties and demonstrate the ability to engineer group I introns for generating circular RNAs as effective vaccines.

## Materials and methods

### Plasmid construction and *in vitro* transcription templates

The 5′ homology domain 1 sequence, 3′ group I intron sequence, IRES sequence, gene of interest (GOI), 5′ group I intron sequence, and 3′ homology domain 2 sequence were polymerase chain reaction (PCR) amplified and cloned into a T7 polymerase-based expression plasmid backbone using a NEBuilder HiFi DNA Assembly kit (NEB) or by a conventional restriction digest and ligation approach. All constructs were confirmed by Sanger sequencing and sequences are provided in the supplementary data. *In vitro* transcription (IVT) templates were prepared by restriction enzyme linearization followed by column clean up.

### Purification of RNase R

To produce the RNase R protein for *in vitro* assay, RNase R from *Escherichia coli* was cloned into a pGEX-6p-1 vector with an N-terminal glutathione S-transferase tag followed by a 3C protease cleavage site. The RNase R construct was expressed in *E. coli* BL21 cells in LB medium at 37°C until an OD_600_ of 0.6–0.8 and induced at 20°C with 0.5 mM Isopropyl ß-D-1-thiogalactopyranoside (IPTG) overnight. Cells were harvested (5000 × *g*, 20 min, 4°C), resuspended in buffer A (20 mM Tris–HCl, pH 7.5, 1 M NaCl, 5% glycerol, 1 mM Dithiothreitol), and lysed by sonication. The lysate was cleared by centrifugation (18 000 × *g*, 1 h, 4°C), and the supernatant was incubated with Glutathione Agarose 4B resin (GE Healthcare). Bound protein was washed with buffer A eluted with buffer B (20 mM Tris–HCl, pH 7.5, 1 M NaCl, 5% glycerol, 1 mM DTT, 50 mM reduced glutathione). The protein was exchanged from buffer to buffer C (20 mM Tris–HCl, pH 7.5, 100 mM NaCl, 5% glycerol, 1 mM DTT) and applied to Heparin HP column (GE Healthcare), eluted with a gradient from 100 mM to 1 M NaCl, and further purified by size exclusion chromatography on a HiLoad 16/600 Superdex 200 pg column (GE Healthcare) in buffer D (20 mM Tris–HCl, pH 7.5, 500 mM NaCl, 5% glycerol, 1 mM DTT).

### Production and purification of circular RNA

About 0.5–1 μg linearized plasmids were used as template for RNA synthesis using HiScribe T7 High Yield RNA Synthesis Kit (NEB) according to the manufacturer’s instructions. Reactions were incubated at 37°C for 2 h and IVT templates were subsequently degraded with 2 μl DNase I (Thermo Fisher) per IVT reaction for 30 min at 37°C. The remaining RNA was then column purified. Subsequently, purified RNA was incubated for 15 min in the following conditions with the addition of a final concentration of 2 mM GTP for circularization: (A) 37°C: 25 mM NaCl, 15 mM MgCl_2_, and 25 mM HEPES (pH 7.5); (B) 55°C: 25 mM NaCl, 15 mM MgCl_2_, 25 mM HEPES (pH 7.5); (C) 37°C: 1 M NaCl, 15 mM MgCl_2_, and 25 mM HEPES (pH 7.5); (D) 37°C: 25 mM NaCl, 200 mM MgCl_2_, and 25 mM HEPES; (E) 37°C: 1 M NH_4_OAc, 100 mM Mg(OAc)_2_, and 25 mM HEPES (pH 7.5); (F) 55°C: 0.5 M KCl, 15 mM MgCl_2_, 40 mM Tris–HCl (pH 7.0), 5 mM DTT, and 2 mM spermidine; and (G) 55°C: 15 mM MgCl_2_, 50 mM Tris–HCl (pH 7.0), and 1 mM DTT. All samples were column purified after folding and circularization. To enrich circular RNA, circularized RNA samples were poly(A) tailed using poly(A) polymerase (NEB) following manufacturer’s protocol. Subsequently, these samples were column purified and treated with home-made recombinant RNase R at 37°C for 30 min, followed by column clean-up. Lastly, these RNA samples were further purified by cellulose to remove double-stranded RNA contaminants [23] and then treated with quick CIP (NEB) to remove phosphates.

To produce circular RNA with modified nucleotides, indicated percentage of *N*^6^-methyladenosine-5′-triphosphate (m6A) (Trilink, N1013), *N*^1^-methylpseudouridine-5′-triphosphate (m1ψ) (Trilink, N1081), isoguanosine-5′-triphosphate (iso-GTP) (Trilink, N1099), 2-thiocytidine-5′-triphosphate (2-thio-CTP) (Trilink, N1036), and 5-methylcytidine-5′-triphosphate (m5C) (Trilink, N1014), and N^4^-acetyl-cytidine-5′-triphosphate (ac4C) (Jena Bioscience, NU-988) substitutes its respective nucleotides in the IVT reactions. Once these RNA samples are produced, they are processed as described above to make circular RNA.

### Production and purification of linear RNA

Linear mRNA is synthesized using the HiScribe T7 High Yield RNA Synthesis Kit (NEB) with the following modifications: CleanCap AG (3′-OMe) (Trilink, N7413) was added to a final concentration of 4 mM and m1ψ (Trilink, N1081) was fully substituted for UTP. Subsequently, these IVT products are purified by Monarch columns (NEB). Next, these RNA samples are further purified using POROS^™^ Oligo(dT) 25 affinity resin (Thermo Fisher) according to the manufacturer’s instructions.

### RNA gel electrophoresis

Agarose gels were prepared by melting RNase-free agarose in Tris–acetate–EDTA buffer with addition of gel red. RNA was denatured in RNA loading buffer (NEB) by diluting 1:1 volumetrically, heating to 65°C for 5 min, and chilling on ice for 1 min. RNA was loaded into each well and run at 100–125 V at room temperature for 45 min. Gels were visualized using Bio-Rad ChemiDoc imaging system. To differentiate circular RNA and nicked circular RNA, RNA samples were denatured with RNA loading buffer (NEB) and were loaded onto 2% E-Gel EX Agarose gels with SYBR-GOLD II (Thermo Fisher). These E-Gel EX gels were run on the E-Gel Powersnap Electrophoresis System using programs ‘EX1-2%’ at room temperature. Images were taken using Bio-Rad ChemiDoc imaging system.

### IRES engineering

Error-prone PCR was performed to randomly introduce mutations into CVB3 IRES using GeneMorph II (Agilent) according to the manufacturer’s instructions. This mutant CVB3 library was subsequently cloned into DNA plasmids for making circular mNeonGreen RNA (circMNG). The circMNG containing CVB3 mutants was transfected into 293T cells. These transfected cells were sorted by fluorescence-activated cell sorting (FACS) by fluorescence intensity (Ex: 488 nm; Em: 510 nm) and sorted cells were harvested for subsequent RNA extraction and nanopore sequencing. Alternatively, these transfected cells were lysed and subjected to polysome fractionation [24]. A total of 12 fractions were collected, and heavy fractions (11 and 12) were collected for subsequent RNA extraction and nanopore sequencing.

### Selection of mutant IRES with higher translation efficiency

The nanopore reads were first demultiplex with Guppy followed by aligning the reads to the CVB3_IRES reference with minimap2 with the default parameters. We next extracted the pileup of reads using SAMtools and used Varscan pileup2snp with the parameters (--min-var-freq 0.001 --*P*-value 0.95) to calculate the mutants in each sample. To identify mutants that are highly enriched, we selected the overlapping the mutants, with *P*-value <.05, in all three samples, and took the mean of the VarFreq (Supplementary Table S2).

### NAI treatment of RNA for structural probing

Eight group I intron (GII) and eight PIE Gaussia luciferase (Gluc) IVT RNAs were resolved by agarose gel, and the largest fragment in each GII and PIE Gluc sample was extracted. These extracted RNAs were separated into three reactions: (i) we added 1:30 the volume of 2 M NAI (03-310, Merck, 1 μl of NAI in 30 μl of RNA in structure buffer (50 mM Tris–HCl, pH 7.4, 10 mM MgCl_2_, 150 mM NaCl) to the RNA and incubated the reaction for 10 min at 37°C for structure probing; (ii) we added 1:30 the volume of dimethyl sulfoxide (DMSO) (D2650, Sigma–Aldrich, 1 μl of DMSO in 30 μl of RNA in structure buffer) to the RNA and incubated the reaction for 10 min at 37°C as a negative control; and (iii) we set aside a third portion of the RNA as for the denaturing control in the downstream library preparation process. We then fragmented all the RNA in MAP fragmentation buffer (18 mM MgCl_2_, 450 mM KCl, 300 mM Tris–HCl, pH 8.3) at 95°C for 1 min 45 s and, followed by RNA purification using RNA Clean & Concentrator-5 (R1014, Zymo) according to the manufacturer’s instructions. We then performed library preparation following the SHAPE-MaP protocol to generate cDNA libraries compatible for Illumina sequencing [25].

### Shape-MaP analysis

After obtaining sequencing data for two biological replicates for each of the eight GIIs and eight PIEs under denaturing conditions and NAI or DMSO treatment, respectively, the reads were aligned with the appropriate reference sequences [26, 27]. Shape reactivity was calculated using shapemapper 2.1.5 [28] with default parameters except for a minimum read depth of 500 instead of 5000 in order to obtain the broadest possible coverage of low-read regions. To identify regions of structural divergence, we plotted the reactivities of the GII versus the reactivities of the PIE for each of the eight specimens. In addition, we calculated Pearson correlation coefficients for the matching GII and PIE segments, which range from 0.65 to 0.82 indicating a high level of structural consistency and suggest that the split of the GIIs into PIEs did not materially disturb the structures.

### Pairwise interactome mapping and SPLASH analysis

Gel-extracted Po and Gv PIE Gluc precursor RNA was denatured at 90°C for 2 min in nuclease-free water and put on ice for 5 min. Subsequently, these RNA samples were folded in structure buffer (50 mM Tris, pH 7.4, 10 mN MgCl_2_, 150 mM NaCl) using a thermocycler with the following program: incubate at 4°C for 2 min, ramp up to 37°C at 0.1°C/s, and incubate 37°C for 30 min. These samples were then treated with biotinylated psoralen, cross-linked at 365 nm, and column cleaned up for SPLASH library preparation as described previously [29].

SPLASH libraries were sequenced using NovaSeq X Plus PE 150. Computational analysis was performed similarly to the published protocol [29]. Briefly, paired-end reads were merged using SeqPrep (v1.3.2). Merged reads were mapped to a reference of Po or Gv PIE Gluc precursor sequence using BWA MEM (v0.7.17). Reads with identical CIGAR strings were considered as duplicates and were collapsed into one read. Primary alignments with the ‘SA’ tag were identified as chimeric reads. Reads that mapped to E1–E2 ligated junction were omitted to rule out reads that the E1–E2 junction was in circular confirmation. Lastly, chimeric reads with mapping quality >20 and arm span >10 bp were used for downstream analysis.

The resultant chimeras were binned into 10-nt windows to quantify the chimeric read count for each window-level pairwise interaction. Interactions with one chimeric read were omitted to reduce background noise. Normalized chimeric count was obtained by dividing chimeric reads against the total mapped reads of the precursor construct. In addition, we analysed the energetics of group I intron RNA duplexes at the observed interaction sites using RNAcofold from the ViennaRNA software package to predict interaction energy, and the base-pairing regions were visualized.

### Determination of exon sequence requirement for circularization

T4 PIE N60 Gluc, An PIE N60 Gluc, Sh PIE N60 Gluc, and Po PIE N60 Gluc IVT RNA were folded in condition B for 15 min or 1 h (55°C: 25 mM NaCl, 15 mM MgCl_2_, 25 mM HEPES, pH 7.5, and 2 mM GTP). These samples were resolved by agarose gel, and the lower band in each sample containing circular RNA was gel extracted. Gel-extracted T4 and An PIE N60 RNA was used for first-strand DNA synthesis using SuperScript III reverse transcriptase (Thermo Fisher) with primer N60 junction R1 according to manufacturer’s instructions, and this complementary DNA (cDNA) was subsequently used as a template for PCR amplification with primers N60 junction F and R1. For gel-extracted Sh and Po PIE N60 RNA, primer N60 junction R2 was used to synthesize first-strand cDNA with SuperScript III reverse transcriptase (Thermo Fisher) and this cDNA was subsequently used as a template for PCR amplification with primers N60 junction F and R2. All these PCR fragments were then processed using NEBNext Ultra II DNA Library Prep Kit to construct libraries compatible for Illumina sequencing. All construct and primer sequences are listed in the Supplementary Table S1. For data analysis, sequencing results were filtered first based on two criteria: (i) The 8-nucleotide flanking sequences besides E1 and E2 are found. (ii) The sequence between the 8-nucleotide flanks is exactly 60 bases in length. These 60-nucleotide sequences were then extracted, deduplicated, and used for downstream sequence context analysis to identify enriched motif sequences.

### Preparation of lipid nanoparticles for RNA encapsulation

The lipid nanoparticles (LNPs) were prepared with the lipids that were used in the Pfizer-BioNTech COVID-19 mRNA vaccine [30]. Briefly, 0.3 mg mRNA was dissolved in 2.25 ml of 1 mM sodium acetate buffer (pH 4.8) (Sigma–Aldrich) to obtain an aqueous phase. A mixture of lipids containing ALC-0315 ([(4-hydroxybutyl)azanediyl]di(hexane-6,1-diol) bis(2-hexyldecanoate)), DSPC (1,2-distearoyl-*sn*-glycero-3-phosphocholine), cholesterol, and ALC-0159 (methoxypolyethyleneglycoloxy(2000)-N,N-ditetradecylacetamide) (all purchased from MedChemExpress, USA) at a molar ratio of 46.3:9.4:42.7:1.6 was dissolved in 0.75 ml of ethanol solution to obtain an organic phase. The N/P ratio of ALC-0315 to mRNA used for mRNA encapsulation was 6. The two phases were mixed by a NanoAssemblr^®^ Ignite microfluidic system (Precision Nanosystems, Canada) at a flow rate ratio of 3:1 (aqueous phase:organic phase) with a total flow rate of 12 ml/min at room temperature to produce LNPs. The formed LNPs were dispersed in 40 ml of saline solution (pH 7.4) for buffer exchange. The diluted LNP suspension was transferred into ultracentrifuge tubes with a molecular weight cut-off of 30 000 Da and centrifuged with 2500 rcf × 4°C × 60 min to remove ethanol and obtain concentrated LNP suspension. The final LNPs were stored at 4°C prior to use.

### Animal study and sera analysis post-vaccination

For mouse vaccination in BALB/c mice, groups of 5- to 6-week-old female mice were intramuscularly immunized with 1 or 5 μg of LNP-RNA, and 3 weeks later, a second dose was administered to boost the immune response. The sera of immunized mice were collected on day 21 (a day before the administration of the second dose) and day 43 (3 weeks after the second dose) for antibody titer measurement and neutralization test. These BALB/c mice were purchased from InVivos and housed under specific pathogen-free conditions in individual ventilated cages. The described animal experiments were approved by the Institutional Animal Care and Use Committee (IACUC) of NUS under protocol number R21-1566 and performed in accordance with the guidelines of the National Advisory Committee for Laboratory Animal Research (NACLAR). Animal facilities are AAALAAC-accredited and licensed by the regulatory body Agri-Food and Veterinary Authority of Singapore (AVA). All efforts were made to minimize animal suffering. For surrogate virus neutralization test (sVNT), the cPass^™^ sVNT (L00847-A; GenScript, USA) was used to measure serum neutralizing antibodies from BALB/against Delta B.1.617.2, whereby the procedure and analysis of neutralizing activity were performed as per manufacturer’s protocol. Serum samples were diluted 1:10. Thirty percent inhibition with this assay is indicated as the cut-off of being considered as positive for neutralizing activity.

### Cell culture and transfection for protein expression analysis

A549, 293T, and HeLa cells were cultured at 37°C and 5% CO_2_ in Dulbecco’s modified Eagle’s medium (Gibco) supplemented with 10% heat-inactivated fetal bovine serum and penicillin/streptomycin (Gibco). RNA delivery was achieved using Lipofectamine MessengerMax reagent (Thermo Fisher) according to the manufacturer’s instructions or by LNP formulations. To determine spike protein expression, 100–200 ng of linearSPIKE or circSPIKE RNA was transfected into cells (5 × 10^4^ cells/well) in a 12-well plate. Approximately 48 h after transfection and at indicated time points, culture medium was collected and analysed by SARS-CoV-2 (COVID-19) Spike RBD protein sandwich ELISA kit (GTX536267, GeneTex). Alternatively, linearSPIKE-HiBit and circSPIKE-HiBit RNA that have a C-terminal HiBit tag were transfected into cells. About 2–3 days after transfection, supernatants were harvested for luminescence measurement using Nano-Glo HiBit Extracellular Detection System (Promega). Both absorbance and luminescence readings were taken using a Tecan Spark multimode microplate reader.

### RNA isolation and real-time quantitative PCR

One hundred nanograms of indicated RNA was transfected into A549 cells (2 × 10^5^ cells/well) in a 12-well plate. Six hours post-transfection, supernatants were removed, and 1 ml Trizol (Thermo Fisher) was added to each well and RNA was isolated according to the manufacturer’s instructions, followed by DNase I treatment to remove genomic DNA. Reverse transcription was performed on 1 μg total RNA using the iScript cDNA synthesis kit (Bio-Rad), and real-time quantitative PCR (RT-qPCR) was performed using SsoAdvanced Universal SYBR Green Supermix reagent (Bio-Rad). RT-qPCR cycling conditions are as follows: 98°C for 1 min, [98°C for 10 s, 60°C for 25 s] (40 cycles). Relative RNA levels of *IFNβ* and *IFIT1* were normalized to *SDHA* and are expressed relative to the control in each experiment. Primer sequences are listed in the Supplementary Table S1.

### RNA pull-down by streptavidin resin

Dynabeads Streptavidin C1 resin (Thermo Fisher) was first washed three times with condition B buffer (25 mM NaCl, 15 mM MgCl_2_, 25 mM HEPES, pH 7.5) and was incubated with Nucleic Acid Detection Blocking Buffer (89880A, Thermo Fisher) at room temperature rotating for 15 min, followed by washing twice with condition B buffer. RNA was folded at 55°C in condition B buffer in the presence of 2 mM GTP and then was cooled down at room temperature for 10 min before mixing with blocked beads in an Eppendorf tube. These tubes were rotated head-to-tail at room temperature for 1 h to allow S1 motif and streptavidin binding. Subsequently, supernatants were removed, and beads were washed four times with SA-RNP wash buffer (300 mM NaCl, 15 mM MgCl_2_, 25 mM HEPES, pH 7.5, 0.02% Tween 20). Bound RNA was eluted with elution buffer (95% formamide, 10 mM EDTA) at 65°C for 5 min and was analysed by electrophoresis.

## Results

### Different group I introns have unique requirements for efficient circularization

**Figure 1.**
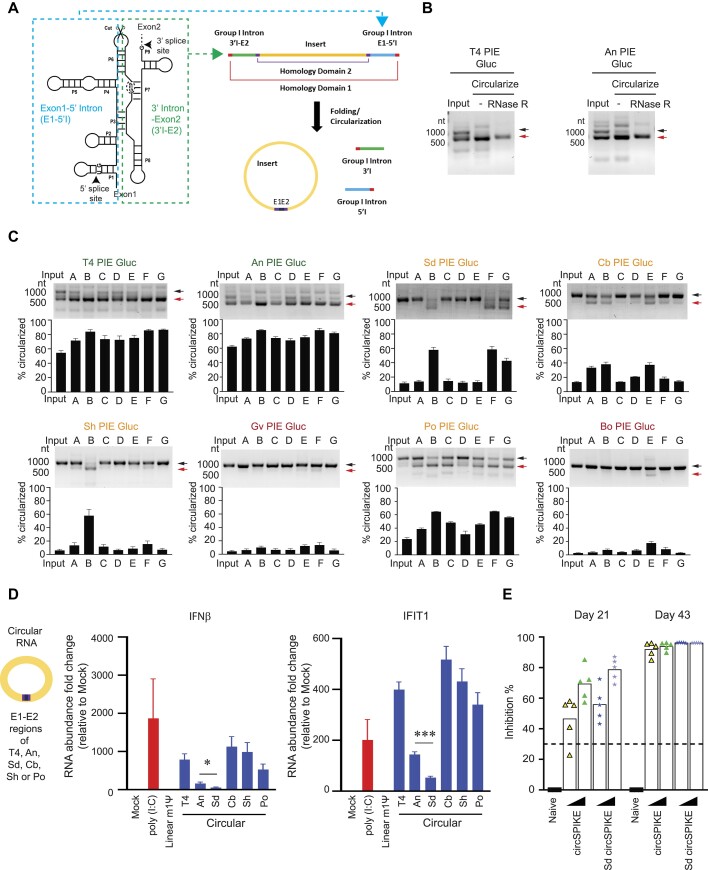
Identification and characterization of novel group I intron PIE for circular RNA production. (**A**) Schematics showing the engineering of group I intron PIE system to make circular RNA. (**B**)IVT RNA precursor was circularized at 55°C in buffers containing 25 mM NaCl, 15 mM MgCl_2_, 25 mM HEPES (pH 7.5). Subsequently, these samples were purified by columns and then treated with RNase R. All these RNA samples were analysed by agarose gel electrophoresis. The black arrow (upper) indicates precursor RNA and red arrow (lower) indicates circularized RNA. (**C**) IVT RNA precursor was folded with indicated conditions and was analysed by agarose gel electrophoresis. Densitometry analysis was performed to quantify the lower band intensity using ImageJ. The black arrow (upper) indicates precursor RNA and red arrow (lower) indicates circularized RNA. Representative gels from three independent experiments are shown. (**D**) The capped linear RNA with 100% *N*^1^-methylpseudouridine (linear m1ψ) that encodes Gluc and indicated circular RNA (circularized at 55°C: 25 mM NaCl, 15 mM MgCl_2_, 25 mM HEPES, pH 7.5) that contains an internal ribosomal entry site CVB3 and Gluc was transfected into A549 cells. Six hours after transfection, total RNA was isolated and *IFNβ* and *IFIT1* transcript abundance was measured by RT-qPCR. Data are mean ± SEM from two independent experiments. Statistical significance was determined using a two-tailed *t*-test: ∗*P*< .05; ∗∗∗*P*< .001. (**E**) Two doses of LNP-circSPIKE or LNP-Sd circSPIKE were injected into BALB/c mice for a dose-response study. Serum samples were collected at 3 weeks after each dose, and neutralizing activity against Delta SARS-CoV-2 was determined. Each dot represents one mouse. The dashed line indicates the cut-off that is being considered as positive for neutralizing activity.

To investigate whether different group I introns can enable RNA circularization upon PIE rearrangement, we tested eight group I intron sequences from different organisms across a range of different buffers, differing in salt and temperature to identify the condition for the most efficient circularization (see the ‘Materials and Methods’ section, Supplementary Table S1). These eight group I introns are from *T4 phage* (T4), *Anabaena* (An), *Scytalidium dimidiatum* (Sd), *Clostridium botulinum* (Cb), *Scytonema hofmanni* (Sh), *Geosmithia virida* (Gv), *Penicillium oblatum* (Po), and *Barrmaelia oxyacanthae* (Bo) [21, 22, 26, 27]. We generated the PIE constructs by splitting the group I introns at the P6 region and moving the right half (3′-intron and exon 2 [3′I-E2]) and the left half (exon 1 and 5′-intron [E1-5′I]) of the intronic sequences to the 5′ and 3′ ends of a gene of interest, respectively (Fig. 1A). If these split group I intron sequences retain their catalytic activities upon splitting, they will cleave and leave behind circular RNAs. As an initial test to confirm whether our PIE systems work, we tested the PIE systems of T4 phage and *Anabaena*, which are well-described PIE systems used for circularizing RNAs. As a proof of concept, we used the Gluc mRNA, a 0.5-kb construct, as a gene of interest between the split group I introns. As expected, we observed efficient circularization of Gluc using PIE systems from T4 phage and *Anabaena* (Fig. 1B). This circular form of the RNA tends to migrate faster in the agarose gel and can be detected in IVT products (input, Fig. 1B). In addition, circularized RNA shows stronger resistance to RNase R (Fig. 1B), indicating that our system generated circular RNAs.

To determine the circularization efficiency of PIEs of other group I introns, T4, An, Sd, Cb, Sh, Gv, Po, and Bo PIE Gluc RNAs were *in vitro* transcribed using T7 RNA polymerase and incubated in various buffer conditions under high temperatures for circularization (buffers A–G, Fig. 1C, Supplementary Fig. S1A, and see the ‘Materials and methods’ section). As expected, T4 and An introns, which have been used extensively in making circular RNAs, showed a high circularization efficiency of above 80% across many different conditions (Fig. 1C). We also observed that Sd, Cb, Sh, and Po introns showed intermediate levels of circularization (∼40%–65%), while Gv and Bo showed poor circularization efficiency of <20% across most buffers (Fig. 1C and Supplementary Fig. S1B). Besides Gv and Bo, most group I intron sequences were able to circularize in at least one buffer condition, and the combination of high monovalent salts, divalent salts, and temperature (55°C, condition B, see the ‘Materials and methods’ section) appears to be the most effective set of elements to enable circularization (Fig. 1C and Supplementary Fig. S1B).

To further determine how the size of a gene of interest may impact circularization, we tested the circularization efficiency of a 1.5-kb firefly luciferase (Fluc), in addition to the 0.5-kb Gluc, using the EX-Gel system. The EX-Gel system has been used in other circular RNA analyses to enable the separation of intact circular RNA versus nicked circular RNA [31]. While the median circularization efficiency of the different group I introns with Gluc (total size 1 kb with 0.5 kb of Gluc gene) was around 60%, we observed a reasonable circularization of ∼40% for group I introns with the larger Fluc (total size 2 kb with insert 1.5 kb) (Supplementary Fig. S1C and D). However, during the process of generating circular RNAs, we also observed a high proportion of nicked RNAs (Supplementary Fig. S1C). As temperature and divalent salts are major factors in breaking RNA, we tested different conditions of Mg^2+^ and temperature to determine whether we could increase the proportion of intact circular RNAs (Supplementary Fig. S2A and B). We observed that a relatively lower concentration of Mg^2+^ (5 mM) decreased the percentage of nicked products during circularization (5 mM versus 15 mM Mg^2+^ at 7 min, or 15 min in buffer B, Supplementary Fig. S2C), suggesting that there is an optimal balance of all parameters to achieve the maximal population of intact circular RNA. Furthermore, digestion of the linear RNA precursor using RNase R post-circularization was able to enrich circular Fluc RNA to above 80% across all group I intron sequences tested (Supplementary Fig. S1E).

To determine whether the circularization of the different group I introns was accurate, we performed deep sequencing around the ligated junction, as well as across the entire circular RNAs, and calculated the frequencies of insertions, mutations, and deletions at these regions (Supplementary Fig. S3A). We observed that most of the circularization events from the different group I introns were highly accurate across the junction, with <1% errors in insertion and mismatch mutations (Supplementary Fig. S3B). Intriguingly, T4 and Po showed a relatively high percentage of deletion rates (>2.5% deletion) in the exon–exon junction during circularization (Supplementary Fig. S3B). However, despite the varying error rates, we observed that the circularization errors occurred within the E1 and E2 domains and did not alter the gene of interest downstream (Supplementary Fig. S3C and D). This suggests that group I intron sequences form highly accurate circles for protein expression. As the rest of the circular RNA outside of the E1–E2 sequences were identical regardless of which group I introns were used for circularization, we utilized nanopore sequencing to sequence the entire circular RNA. Despite the higher intrinsic error rates in nanopore sequencing, we did not observe a significant increase in error rates outside of the gene of interest (Gluc), compared to within the Gluc sequence (Supplementary Fig. S3E), indicating that circularization sequences outside of Gluc are generally accurate. Collectively, these results indicate that in addition to the traditional T4 and anabaena group I introns, other group I introns, namely Sd, Cb, Sh, and Po, can also be designed as PIE systems to generate circular RNAs efficiently and accurately.

### Identification of a group I intron with minimized innate immune activation

Circular RNAs generated from different group I introns can trigger different levels of innate immune activation inside cells. To determine the innate immune response induced by the group I introns, we tested for interferon-beta and IFIT1 expression in A549 cells after the different circular RNAs were transfected into them (Fig. 1D). We observed that circular RNAs generated using T4 phage induced a high level of interferon-beta and IFIT1 expression inside cells as compared to capped, modified*N*^1^-methylpseudouridine (m1ψ), and poly(A)+ tailed linear mRNA, as previously described in the literature [3]. Additionally, circular RNAs generated from *Anabaena* showed a lower level of induction of interferon-beta and IFIT1 compared to T4 phage (Fig. 1D) [32]. Interestingly, we observed that circular RNAs generated using Sd demonstrated three times lower interferon-beta induction compared to circular RNAs generated using An (Fig. 1D), suggesting that Sd could be used to generate circular RNAs while keeping unwanted innate immune activation to a minimum, which is ideal in gene replacement therapies. Additionally, the circular RNAs generated using Cb, Sh, and Po showed generally high reactivities, with Cb inducing an even higher amount of interferon-beta and IFIT1 expression than circular RNAs generated from T4 phage (Fig. 1D).

In addition to mRNAs, circular RNAs have been shown to be able to act as effective vaccines [9]. To confirm whether our circular RNAs can function as good vaccines, we first compared the protein levels of SARS-CoV-2 delta spike expressed from capped, m1ψ, and polyA tailed linear mRNA (linearSPIKE) and from a circular RNA that has a coxsackievirus B3 (CVB3) IRES and is made from An group I introns (circSPIKE). We observed that, compared to linearSPIKE, circSPIKE produced higher amounts of delta SPIKE protein over time in HeLa cells (Supplementary Fig. S4A–D). We then encapsulated linearSPIKE and circSPIKE using LNPs and injected 100 ng of the formulated RNAs into two groups of mice (Supplementary Fig. S5A). As expected, both linearSPIKE and circSPIKE generated high amounts of neutralizing antibodies in mice (Supplementary Fig. S5B), although circSPIKE induced higher levels of neutralizing antibody at an early time point of 7 days (Supplementary Fig. S5B). Additionally, sacrificing and harvesting the mice for their T cells at 7 weeks post-boost also showed robust CD4 and CD8 T cell responses (Supplementary Fig. S5C), confirming that circSPIKE and linearSPIKE can activate both the B cell and T cell response in mice.

To test whether our panel of group I introns can induce strong immunogenicity in mice, we encapsulated Sd circSPIKE made using Sd group I intron using LNP and compared its vaccine efficacy with formulated circSPIKE made from An group I introns. We injected two doses of 1 and 5 μg of encapsulated circSPIKE and Sd circSPIKE into mice. Though both circSPIKE and Sd circSPIKE induced strong neutralizing activity after two doses, interestingly, we observed that Sd circSPIKE resulted in slightly higher neutralizing activity 21 days after dose 1 (Fig. 1E). In conclusion, Sd circSPIKE can be used as an effective RNA vaccine candidate.

### Incorporation of modified nucleotides reduces innate immune activation and enhances translation of circular RNAs

The current SARS-CoV-2 mRNA vaccines are 100% modified with m1ψ, which reduces the innate immune activation of mRNAs and increases their protein production inside cells [3]. While incorporating a small amount of m6A (5%–10%) into circular RNAs has been shown to reduce innate immune activation of circular RNAs [33], it is unclear how and whether other types of modifications can help avoid triggering innate immune activation and promote protein production in circular RNAs. To address this, we tested six different modifications, including m6A, m1ψ, m5C, isoGTP, 2-thio-CTP, and ac4c, and incorporated these modifications individually at different concentrations into circSPIKE-HiBit. A HiBit tag was included at the C-terminus to test protein expression by luminescence measurement. We chose these modifications because they are common RNA modifications and cover all four bases, and because several of them have been shown to improve translation [34–36]. We observed that none of the modifications affected IVT yields for circSPIKE-HiBit until we doped in 50% of the modified nucleotides, although higher modifications of m6A and m1ψ at 50% were found to abolish circularization of circSPIKE-HiBit (Supplementary Fig. S6A and B). This observation is consistent with the understanding that the RNA structure of the group I introns is important for circularization and that the modifications likely impact the proper folding of these group I introns.

**Figure 2.**
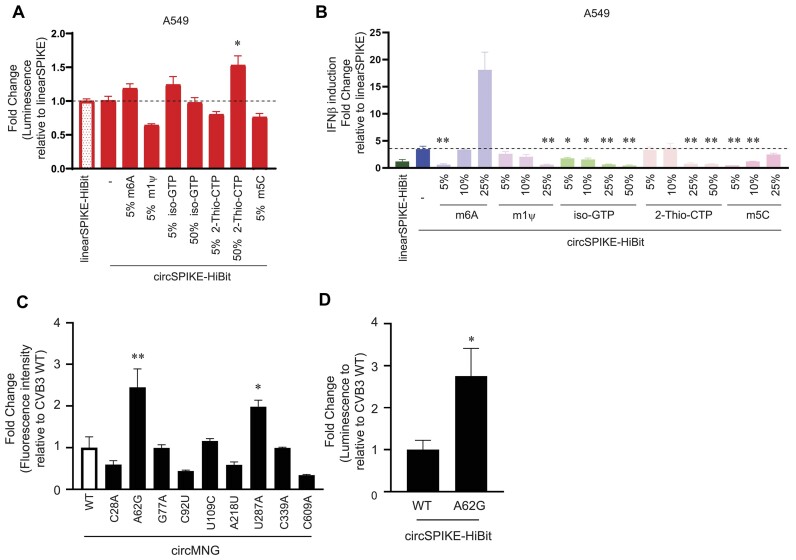
Incorporation of modified nucleotides in circular RNA reduces reactogenicity and IRES engineering increases protein production. (**A**) A549 cells were transfected with linearSPIKE-HiBit that has a cap and 100% *N*^1^-methylpseudouridine and with circSPIKE-HiBit (circularized at 55°C: 15 mM MgCl_2_, 50 mM Tris–HCl, pH 7.0, and 1 mM DTT) with or without various indicated modifications. Approximately 72 h post-transfection, cellular supernatants were harvested for luminescence measurement to assess protein expression. (**B**) Indicated RNA was transfected into A549 cells. About 6 h post-transfection, cells were harvested for RNA extraction, and IFNβ transcript abundance was determined by RT-qPCR. Data are presented as fold change relative to linearSPIKE-Hibit, and error bars indicate SEM. Each experiment was performed two or three times independently. Statistical significance was determined using a two-tailed *t-*test comparing circSPIKE-Hibit with and without modifications: ∗*P*< .05 and ∗∗*P*< .01. (**C**) 293T cells were transfected with circMNG containing CVB3-WT or indicated mutations. About 2 days post-transfection, cells were analyzed by flow cytometry, and data were presented as fold change relative to CVB3-WT. Statistical significance was determined using ANOVA: ∗*P*< .05 and ∗∗*P*< .01. (**D**) circSPIKE-Hibit containing CVB3-WT or CVB3-62G was transfected into 293T cells and luminescence was measured to assess protein expression. Each experiment was performed three times independently and data are presented as fold change relative to CVB3-WT and error bars indicate SEM. Statistical significance was determined using a two-tailed *t*-test: ∗*P*< .05.

Intriguingly, we did not observe that incorporating RNA modifications increased the protein production of circSPIKE-HiBit in HEK293T cells (Supplementary Fig. S6C and D), although adding 5% m6A, 5% isoGTP, and 50% 2-thio-CTP in circSPIKE-HiBit did mildly increase protein production in A549 cells (Fig. 2A). Additionally, increasing the concentration of doped in modified nucleotides generally resulted in decreased protein production in cells, again suggesting that the structure of the IRES of circSPIKE-HiBit might be affected by the modifications, thus impacting its ability to recruit ribosomes for cap-independent translation (Fig. 2A and Supplementary Fig. S6C and D). Of the tested modifications, only 2-Thio-CTP was found to enable high protein production at high concentrations of incorporation (50%, Fig. 2A, Supplementary Fig. S6C). Importantly, although the modifications had a mild impact on the translation of circSPIKE-HiBit, most of them decreased the amount of interferon-beta expression induced by the circular RNA (Fig. 2B). Incorporating 5% of m6A, 25% of m1ψ, >5% of iso-GTP, >25% of 2-thio-CTP, and 5%–10% of m5C decreased the amount of interferon-beta expression of the circSPIKE-HiBit to below that of unmodified circSPIKE. In particular, the interferon-beta induction of several of these modified circular RNAs was almost as low as that of the 100% m1ψ-modified linearSPIKE-HiBit (Fig. 2B). Taken together, these data indicate that 2-thio-CTP could potentially be added to circular RNAs to increase protein production and to reduce unwanted innate immune activation.

### IRES engineering to increase protein production from circular RNA

As the ability of a circular RNA to produce a protein of interest depends on the recruitment of ribosomes by its IRES, we sought to enhance protein production from circular RNA by engineering the IRES. To this end, random mutations were introduced by error-prone PCR into CVB3, and this CVB3 mutant library was cloned into constructs to make circMNG (see the ‘Materials and methods’ section). The circMNG containing CVB3 mutants was transfected into 293T and FACS sorting or polysome fractionation was performed to isolate CVB3 mutants showing higher translatability (see the ‘Materials and methods’ section, Supplementary Table S2). Interestingly, among the selected CVB3 mutants for validation, A62G and U287A showed a two-fold increase in protein production (Fig. 2C and Supplementary Fig. S7) and this enhancement was also observed in circSPIKE-HiBit (Fig. 2D). Taken together, these findings suggest that CVB3 A62G increases protein production in a gene-independent manner, which demonstrates the feasibility of this platform for engineering stronger IRES.

### Group I introns that circularize well show disruptions in P9 structures

**Figure 3.**
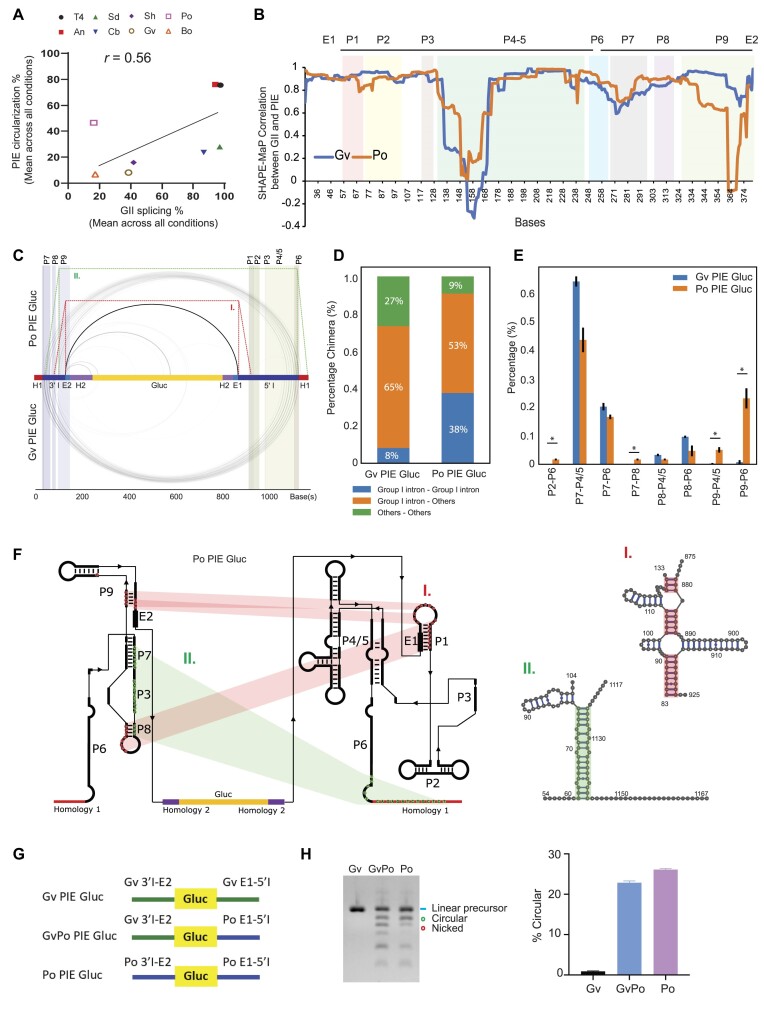
Structural mapping of GII and PIE RNA. (**A**) A scatter plot showing the correlation between PIE circularization and GII splicing from different group I intron sequences. (**B**) Comparison of Gv and Po in SHAPE-MaP correlation between GII and PIE. (**C**–**E**) Mapping of intramolecular interactions in Po PIE Gluc and Gv PIE Gluc. Data are presented in arc plots and bar graphs showing interaction types. H1 and H2 indicate homology 1 and homology 2, respectively. Differential analysis was performed using an independent student’s *t*-test: ∗*P*< .05. (**F**) Key interactions in Po PIE Gluc and base-pairing regions from RNAcofold analysis are highlighted in red (I) and green (II). (**G**, **H**) Indicated constructs were IVT and their IVT products were analysed by E-gel EX electrophoresis. The green circle indicates circular RNA. The blue line indicates IVT RNA precursor, and the red circle indicates nicked circular RNA. Each experiment was performed two times independently and representative gel images are shown.

Different group I introns showed differences in their efficiency of circularization versus efficiency of splicing, with Gv, Cb, and Sd showing poorer circularization and Po showing better circularization than one would expect based on their ability to self-splice (Fig. 3A and Supplementary Fig. S8). However, the mechanistic details underpinning these differences in efficiencies are poorly understood. We reason that structure-based interrogation can reveal critical features for efficient circularization. In line with this, our mutational analysis of another group I intron, *A. tumefaciens* (At), showed that modulating the P9 (At mut4, by deleting part of the P9 domain) and P6 domains (At mut5, by extending the P6 domain) to resemble An (Supplementary Fig. S9A and B) can reduce or enhance circularization efficiency, respectively (Supplementary Fig. S9C and D). These findings prompt us to perform a more thorough RNA secondary structure probing to understand why some group I introns circularize or splice better.

We confirmed that group I introns in the GII or PIE forms utilize similar mechanisms for splicing and for circularization, respectively, by introducing GII splicing-defective mutations in the P7 region [37] into the PIE system to test their effects on circularization (Supplementary Fig. S10A). Indeed, mutations in the P7 region of the T4 group I intron were found to abolish self-splicing (Supplementary Fig. S10B and C) and impair PIE circularization (mut1 and mut2, Supplementary Fig. S10D and E), and the restoration of this base-pairing (mut3) partially rescued splicing and circularization (Supplementary Fig. S10B–E), suggesting that T4 PIE circularization and T4 GII splicing share similar catalytic mechanisms.

We then performed structure probing using SHAPE-MaP on the group I introns in their original sequence (GII) and in the split PIE system. We observed that most of the group I introns showed moderate to high levels of correlation (*R*> 0.65) in their SHAPE-reactivities between the native GII and split PIE from (Supplementary Fig. S11A and B, and Supplementary Table S3), indicating that the two forms generally fold into similar structures despite differences in circularization (Fig. 3A). To determine whether there are specific structural regions that show greater differences between GII and PIE systems that could contribute to differences in circularization abilities, we performed Pearson correlation of the SHAPE reactivities of the GII and PIE systems for each of the P1–P9 domains of the different group I introns (Supplementary Fig. S12). We observed the poorest correlation between GII and PIE around the P7 domain, wherein the nearby PIE cleavage probably results in a loss of pairing and increased structure rearrangement of adjacent sequences (Supplementary Fig. S12).

As the P3–P7 region is important for the catalytic activities of the group I introns, we checked whether preserving these structures was associated with increased circularization. Across the P1–P9 domains, we observed that the more similar structures in the P7 domain between PIE introns and GII resulted in better circularization efficiency, confirming that the P7 structure is important (Supplementary Fig. S13). Across the other regions, we observed the strongest association between structural differences in the P2 and in the later parts of P9 domains of the GII and PIE system with circularization efficiency. Larger differences between GII and PIE reactivities in the P2 and P9 domains were associated with better circularization (Fig. 3B and Supplementary Fig. S13), suggesting that extensive refolding in these two domains occurs in the PIE system. As L2.1 is known to form long-range interactions with L9.1 in group I introns such as *Tetrahymena* [38], we hypothesize that separating L2 and L9 could result in the formation of alternative structures that could support or hinder circularization.

To deepen our understanding of how RNA structure impacts circularization, we examined the structure profiles of two group I introns (Gv and Po) that have similar sequences but different abilities to circularize (Fig. 1C). Gv and Po have high sequence similarity in phylogenetic analysis (Supplementary Fig. S14) and can both undergo splicing [26] (Supplementary Fig. S8). Yet, Po can circularize much better than Gv (Fig. 3A). Aligning the structural similarity between GII and PIE for Po and Gv, we observed that Po showed a much stronger disruption in RNA structure in the P9 and P2 regions than Gv (Fig. 3B), consistent with the general importance of the P9 region that we observed above.

In addition to determining which bases are paired or unpaired, we were curious as to how the bases are paired to each other in the Po and Gv PIE precursor forms. To do this, we performed proximity ligation sequencing using SPLASH on the Po and Gv PIE systems (see the ‘Materials and methods’ section, Supplementary Table S4), which showed long-range interactions between the two halves of the PIE system even when they are separated from each other (Fig. 3C). Interestingly, out of all observed pairwise RNA–RNA interactions, interactions in the Po PIE system tended to reside more between different domains of the group I intron as compared to interactions in the Gv PIE system, which tended to reside between the group I intron and other sequences, such as the luciferase sequence or within the luciferase sequence itself (Fig. 3D). To identify the interactions that are stronger in Po than Gv and that could be associated with increased circularization, we performed a differential analysis and observed that the P9 region showed the largest and most consistent increase in interactions in Po as compared to Gv (Fig. 3E and Supplementary Fig. S15A). To visualize the potential pairwise interactions between the regions, we then performed CoFold analysis on these Po and the corresponding Gv pairings. Interestingly, we observed good RNA–RNA pairwise interactions between P8/P9 and P1 domains, as well as between the P8 and P6 regions for the Po introns, while the same pairwise RNA–RNA interactions in the Gv intron tended to be poor (Fig. 3F and Supplementary Fig. S15B). This suggests that these interactions could bring the two halves of the group I intron into close proximity for circularization.

To test whether increased pairing between the two halves indeed improves circularization, we swapped the second half of Gv with the second half of Po to generate a chimeric GvPo sequence (Fig. 3G). Interestingly, switching the second half of Gv with Po (GvPo) restored the pairwise RNA–RNA interaction (Supplementary Fig. S15C) and improved RNA circularization to similar levels as Po circularization (Fig. 3H). This suggests that understanding the structural basis of group I intron PIE systems can enable us to engineer group I introns with better circularization efficiencies.

### E1–E2 regions can tolerate sequence changes to insert a purification tag for improved manufacturing

**Figure 4.**
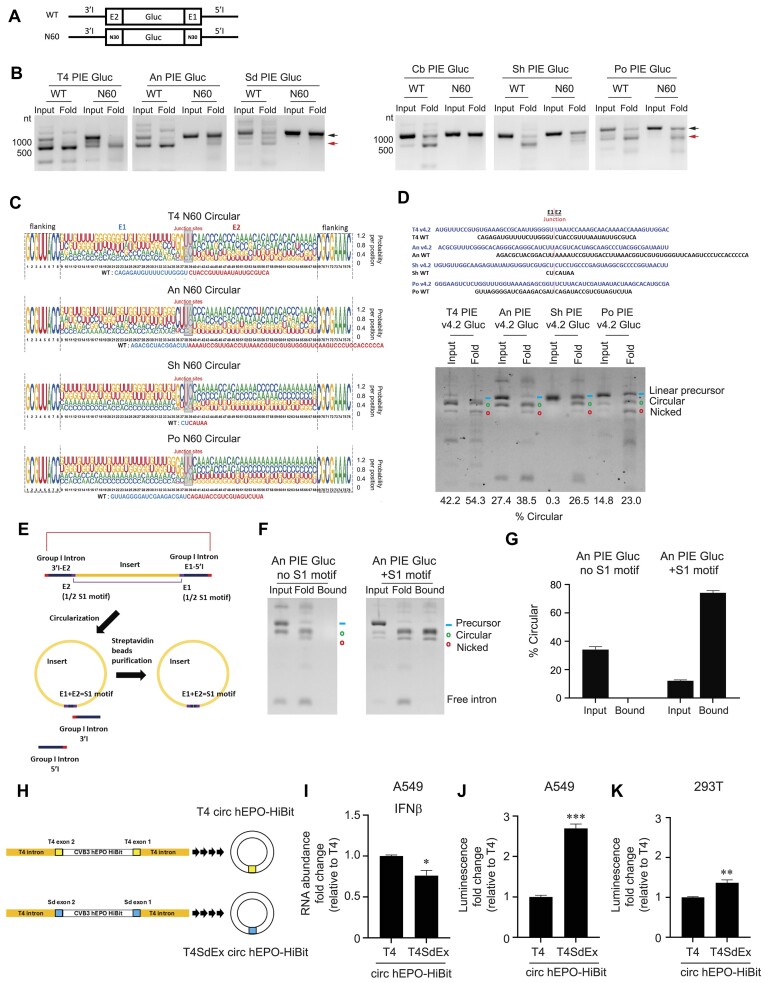
Characterization of E1 and E2 sequence requirement for PIE circularization. (**A**) A schematic showing WT and N60 constructs used in these experiments. N represents randomized nucleotide. (**B**) Indicated IVT RNA was circularized at 55°C in buffers containing 25 mM NaCl, 15 mM MgCl_2_, and 25 mM HEPES, pH 7.5 and column purified. The resulting samples were analysed by agarose gel electrophoresis. The black arrow (upper) indicates precursor RNA and the red arrow (lower) indicates circularized RNA. (**C**) Junction sequences of circular T4, An, Sh, and Po PIE N60 Gluc RNA. (**D**) IVT products and folded RNA of T4, An, Sh, and Po PIE v4.2 Gluc were analysed by E-Gel EX electrophoresis. (**E**) A schematic showing an engineered S1 motif for circular RNA purification. (**F**, **G**) An PIE Gluc RNA with or without S1 was folded and mixed with streptavidin C1 beads. After washing and elution, bound fractions were analysed by E-Gel EX electrophoresis. Green circle: circular RNA. Blue line: IVT RNA precursor. Red circle: nicked circular RNA. Each experiment was performed two or three times independently and representative gel images are shown. (**H**) Schematics of T4 and T4SdEx circ hEPO-Hibit. (**I**–**K**) Circular T4 and T4SdEx RNAs encoding CVB3 and hEPO-Hibit were transfected into A549 cells. Six hours after transfection, total RNA was isolated, and *IFNβ* transcript abundance was measured by RT-qPCR. For assessing protein expression, supernatants from transfected A549 and 293T cells were collected for luminescence measurement 72 h post-transfection. Data are mean ± SEM from two independent experiments and are presented as fold change relative to T4. Statistical significance was determined using a two-tailed *t*-test: ∗*P*< .05; ∗∗*P*< .01; ∗∗∗*P*< .001.

As E1 and E2 sequences typically leave behind a 20–50-nucleotide scar within the circularized RNA, we investigated whether there are sequence determinants for the E1 and E2 sequences for each group I intron and whether there could be opportunities for engineering these E1 and E2 sequences. To test whether there are sequences in E1 and E2 domains that can impact the circularization of different group I intron PIE systems, we synthesized randomized E1 and E2 sequences by replacing the original sequences with 30-Ns on each side of Gluc to determine the nucleotide features that drive circularization (Fig. 4A). Upon generating the randomized E1 and E2 sequences, we circularized these RNAs under high salt and temperature conditions (condition B: 25 mM NaCl, 15 mM MgCl_2_, 25 mM HEPES, pH 7.5, and 2 mM GTP at 55°C for 15 mins see the ‘Material and methods’ section) and ran them onto an agarose gel. Interestingly, we observed that circular RNA products can be generated using randomized E1 and E2 sequences for T4, An, Sh, and Po group I introns, indicating that their E1 and E2 sequence contexts can be flexible (Fig. 4B). In contrast, the E1 and E2 sequences for Sd and Cb PIE circularization appear to be more sequence-specific despite having similar lengths, as we could not detect circularization for them even under long incubation times, upon randomizing their sequences (Fig. 4B and Supplementary Fig. S16).

To determine what these other E1 and E2 sequences that enable circularization for T4, An, Sh, and Po group I introns are, we gel extracted the circularized RNAs and cloned them into individual cDNA libraries for deep sequencing (Supplementary Fig. S16). Our sequencing analysis showed that the base at the E1/E2 junction (U), as well as the base immediately upstream and downstream of the junction, was conserved, while the rest of the bases were able to take on different sequences for circularization (Fig. 4C). To test this, we engineered completely new E1–E2 sequences for these four group I introns and showed that the new sequences can indeed enable circularization (Fig. 4D). This suggests that E1 and E2 domain sequences are amenable for engineering for properties of interest.

One of the main challenges in using circular RNAs in medicine is purifying them efficiently. While one can perform positive selection on linear mRNAs using oligo(dT) beads, it is more challenging to perform a negative selection to purify circular RNA away from its linear precursor RNAs and spliced introns. As such, we were curious as to whether including elements in the E1–E2 domain could enable a positive selection of circular RNAs. To this purpose, we inserted halves of the streptavidin binding S1 motif into the E1 domain and E2 domain, such that the halves could form a complete S1 motif upon circularization (Fig. 4E). This complete S1 motif would then allow purification of the circularized RNAs using streptavidin beads. Interestingly, we observed that circular RNAs with S1 motifs in the E1–E2 sequences were enriched in the total RNA population upon binding to streptavidin beads, unlike the result without streptavidin purification or circular RNAs without S1 motifs (Fig. 4F and G). These results indicate that motifs such as S1 could be incorporated into circular RNAs for downstream purification to enable circular RNAs to be positively selected like linear mRNAs for improved manufacturing.

As the E1–E2 sequences that are incorporated into circular RNA appear to determine the level of innate immune activation by these RNA molecules (Fig. 1D), we tested whether we could engineer E1–E2 sequences for reduced innate immune activation to increase protein production. We generated a chimeric T4SdEx sequence that contains T4 group I intron sequences and Sd exon sequences. Circular RNAs encoding a CVB3 IRES and the human erythropoietin (hEPO) gene fused with a HiBit tag were produced using T4 sequences (T4 circ hEPO-Hibit) or T4SdEx sequences (T4SdEx circ hEPO-Hibit), respectively (Fig. 4H and Supplementary Fig. S17A and B). Both circular RNAs were transfected into cells to assess their potency to activate innate immune response and their ability to produce proteins inside cells. Interestingly, T4SdEx circ hEPO-Hibit induced a lower level of interferon-beta expression and produced roughly three times the amount of protein compared to T4 circ hEPO-Hibit (Fig. 4I–K). These results demonstrate that E1–E2 sequences can be engineered to incorporate less immunostimulatory sequences to reduce the unwanted innate immune activation by circular RNAs for enhanced protein production as RNA vaccines and therapeutics.

## Discussion

While group I introns have been extensively used to generate circular RNAs, the mechanisms by which they perform efficient circularization are still largely unknown. In this study, we investigated the properties of eight group I introns, including their structure and sequence contexts for making circular RNAs. We observed that rearrangement of the P9 structure is important for efficient circularization of RNA, and we found that swapping sequences can improve the circularization efficiency of poor circularizers such as Gv.

The amount of reactogenicity induced by RNA drugs is a key consideration in the development of RNA vaccines and therapeutics, with reactogenicity showing an association, at least in part, with dysregulated and unwanted innate immune activation [39, 40]. Here, we observed that circular RNAs made by different group I introns generate different levels of innate immune gene expression inside cells. This is important as high innate immune activation of circular RNAs can potentially enable them to act as adjuvants, while low innate immune activation of circular RNAs is usually desired in gene replacement therapy. Out of the six new group I introns, Cb was found to induce the highest interferon-beta and IFIT1 expression, greater even than that of T4, while Sd showed the lowest level of interferon-beta induction in the panel (three times lower than An). As a proof of concept, our study showed that circular RNAs generated using either An or Sd group I introns and a 5-kb insert, including a CVB3 IRES and full-length spike of SARS-CoV-2 delta variant, can be circularized and can successfully induce a robust immune response against SARS-CoV-2. The broad range of potency in activating innate immunity in different group I introns can be a useful feature in selecting which group I intron to use for generating circular RNA vaccines or therapeutics.

Additionally, by using a randomized library for screening, we found that E1 and E2 sequences are flexible for T4, An, Sh, and Po PIE systems, allowing us to change these E1–E2 sequences to incorporate sequences with properties of interest. We showed two applications of this, one using a split S1 motif for purification of circular RNAs and another incorporating less immunostimulatory sequences to avoid unwanted innate immune activation. We believe that other sequences and structural motifs of interest can also be incorporated into the E1–E2 sequences to design circular RNA with different properties of interest.

In summary, durable protein expression and extended antigen presentation may give circular RNA an edge for future vaccine development [41, 42]. Here, we showed that circular RNAs encoding full-length SARS-CoV-2 spike with An and Sd intron PIE systems robustly induce protective antibody. The rules of E1–E2 sequences, in addition to RNA structural rules between the group I intron regions, enable us to engineer new sequences that can confer additional properties for circular RNAs and can accelerate the application of circular RNA in vaccines in the near future.

## Supplementary Material

gkaf089_Supplemental_Files

## Data Availability

Sequencing data presented in this manuscript are publicly available in the GEO repository (GSE239954 and GSE277434).
