# Supplementary material for: Characterization of group I introns in generating circular RNAs as vaccines
Source: Nucleic Acids Res. 2025 Feb 28;53(4):gkaf089. doi: 10.1093/nar/gkaf089 (PMC11879131; doi:10.1093/nar/gkaf089)
Supplement: gkaf089_Supplemental_Files [file gkaf089_supplemental_files.zip › 250114_supp_figures_and_materials.pdf]

Supplementary Figure 1

a

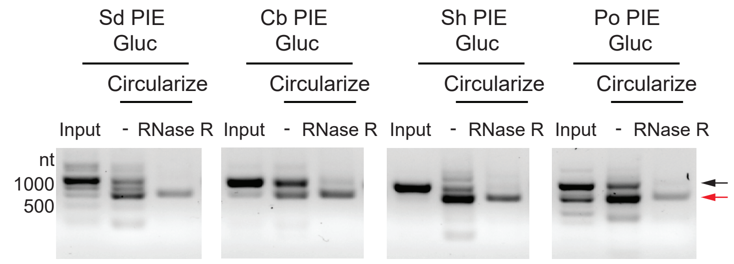

b

% Lower RNA (Mean±SEM)	Conditions							
	Input	A	B	C	D	E	F	G
T4 PIE Gluc	54.6±3.1	71.6±3.0	83.9±2.9	73.6±5.0	72.6±5.3	75.2±3.8	85.7±1.6	86.5±1.0
An PIE Gluc	62.3±1.6	73.4±1.0	85.3±0.6	74.3±1.9	70.9±2.6	75.3±2.3	85.1±2.6	80.9±1.7
Sd PIE Gluc	11.4±1.7	13.9±1.8	58.0±3.4	14.5±2.8	12.2±2.1	12.9±2.0	58.6±4.3	42.6±3.8
Cb PIE Gluc	13.4±0.9	33.4±2.1	38.1±3.1	13.7±0.3	20.9±0.5	37.4±3.0	18.2±2.2	14.4±1.2
Sh PIE Gluc	6.1±1.7	13.4±4.1	58.0±9.4	11.7±3.2	6.5±1.5	8.7±2.8	15.5±4.6	7.0±2.0
Gv PIE Gluc	4.4±1.5	6.1±1.8	10.0±2.1	6.5±1.8	6.4±2.1	12.3±1.8	13.7±3.8	5.7±2.0
Po PIE Gluc	23.6±2.3	38.6±1.6	64.5±0.5	48.4±1.4	30.8±4.8	45.5±1.7	65.1±0.5	56.3±0.8
Bo PIE Gluc	2.8±0.6	3.9±0.8	7.4±1.5	4.1±1.2	6.7±1.6	17.7±2.4	8.5±2.9	3.1±0.9

70-100%	High
30-70%	Intermediate
0-30%	Low

c

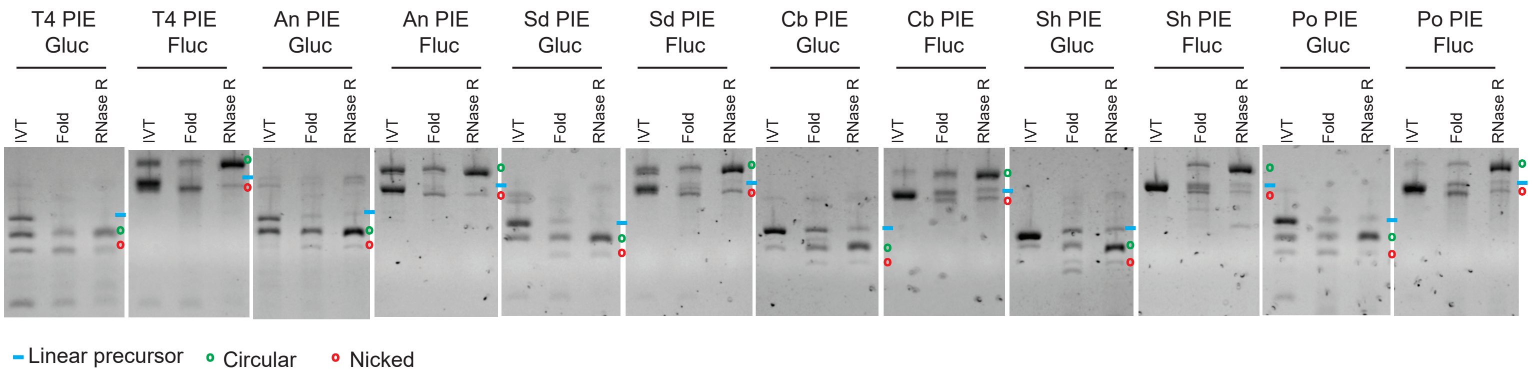

d

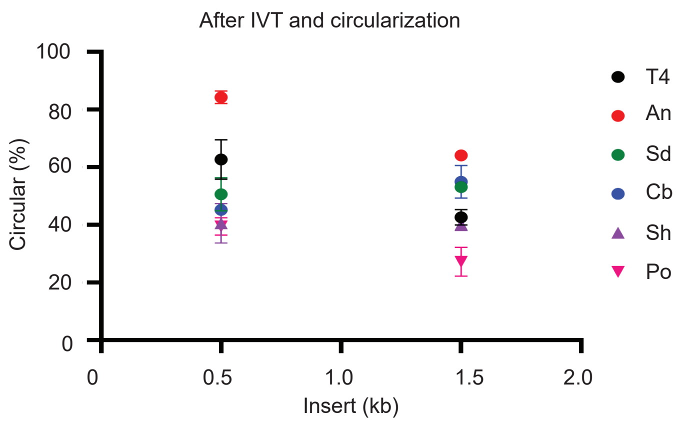

e

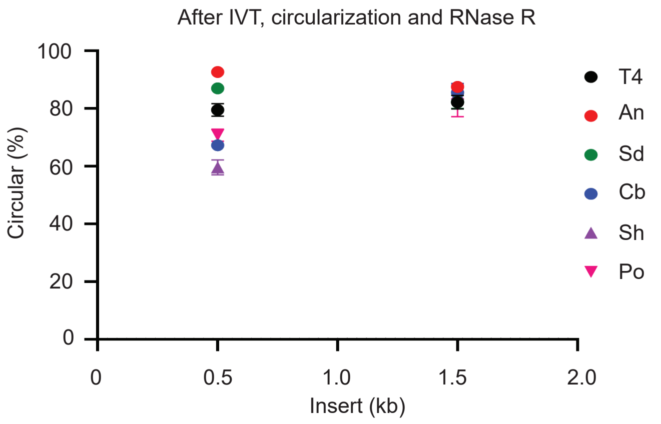

Figure S1. Characterization of novel group I intron PIE for circular RNA production.

(A) In-vitro transcribed (IVT) RNA precursor was folded, circularized (at 55°C – 25mM NaCl, 15mM $MgCl_2$, 25mM HEPES pH7.5) and then treated with RNase R for circular RNA enrichment. All these RNA were analyzed by agarose gel electrophoresis. Black arrow indicates RNA precursor and red arrow indicates circularized RNA. (B) Using densitometry analysis to quantify lower band in different group I intron PIE RNA samples folded under various conditions. (C)(D)(E) T4, An, Sd, Cb, Sh, Po PIE Gluc and Fluc RNAs were in-vitro transcribed, folded in a buffer containing 25mM NaCl, 15mM $MgCl_2$, 25mM HEPES pH7.5 at 55°C, and treated with RNase R. All these samples were analyzed by E-gel EX electrophoresis and densitometry analysis was performed to quantify the circular RNA band intensity. Data are mean ± S.E.M from two or three independent experiments, and representative images are shown.

Supplementary Figure 2

a

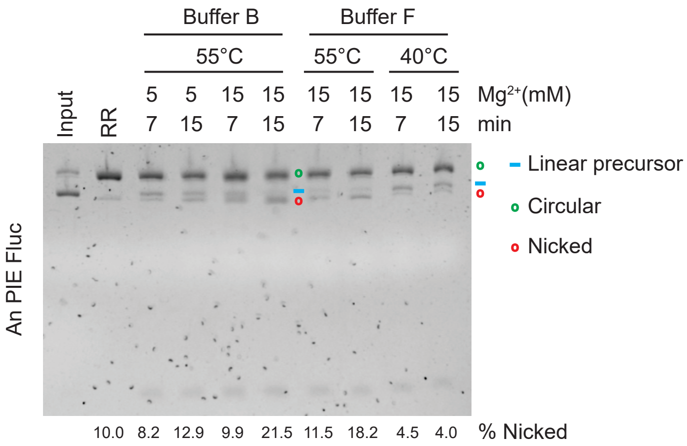

b

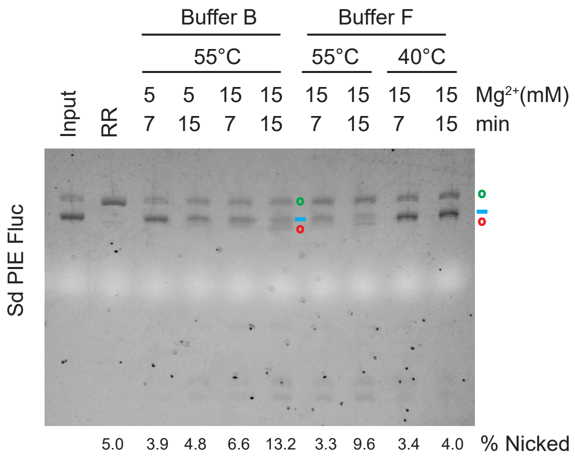

c

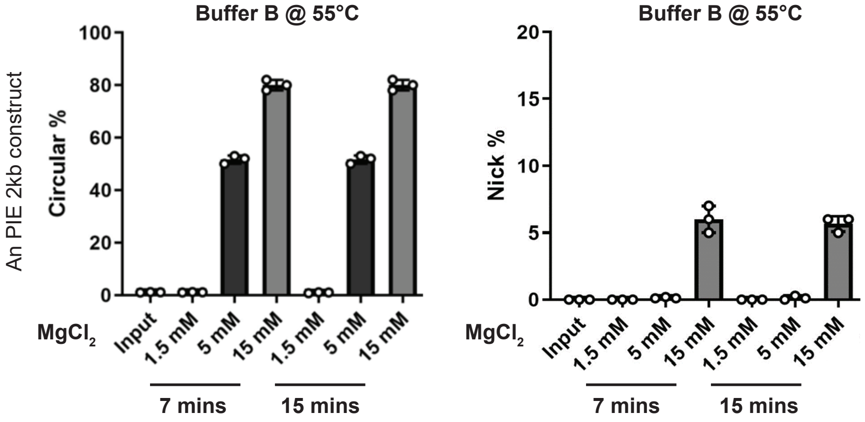

Figure S2. Testing different conditions for circularization.

(A)(B) Optimization of An and Sd PIE Fluc circularization. (C) The effect of folding duration and $MgCl_2$ concentration on circularization efficiency and nicked RNA production. Buffer B contains 25mM NaCl, 25mM HEPES pH 7.5, and $MgCl_2$ at indicated concentrations. Buffer F contains 0.5M KCl, 40mM Tris-HCl pH 7.0, 5mM DTT, 2mM Spermidine and $MgCl_2$ at indicated concentrations.

Supplementary Figure 3

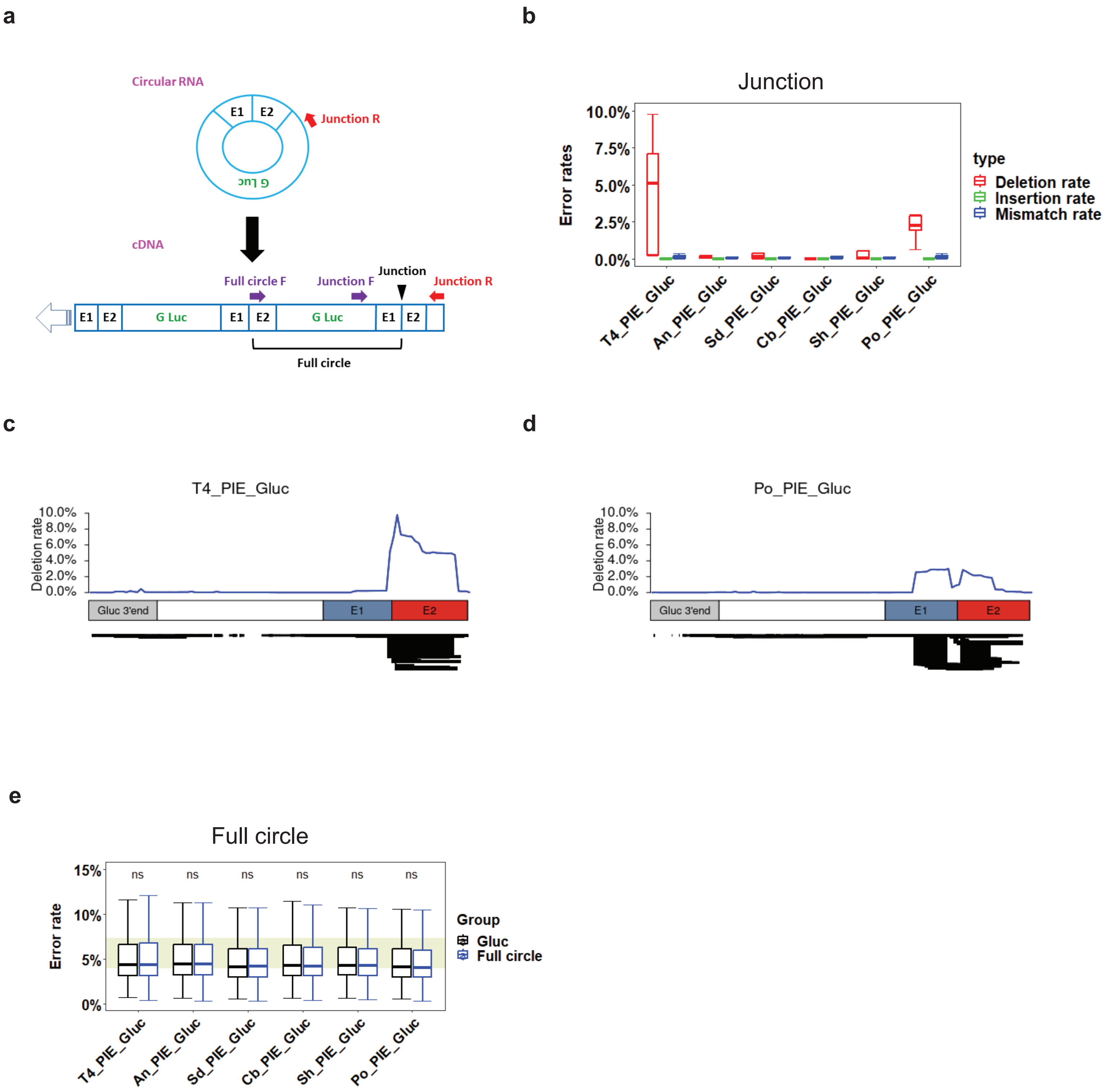

Figure S3. Junction accuracy analysis.

(A) A schematic showing the experimental outline for junction and full circle accuracy analysis. (B) Error rates of the junction area in circular RNA. (C)(D) Deletion distribution in the junction area of T4 and Po circular RNA. (E) Error rates of the whole circular RNA. Green areas indicate Nanopore R9.4.1 error rate range. ns: not significant.

Supplementary Figure 4

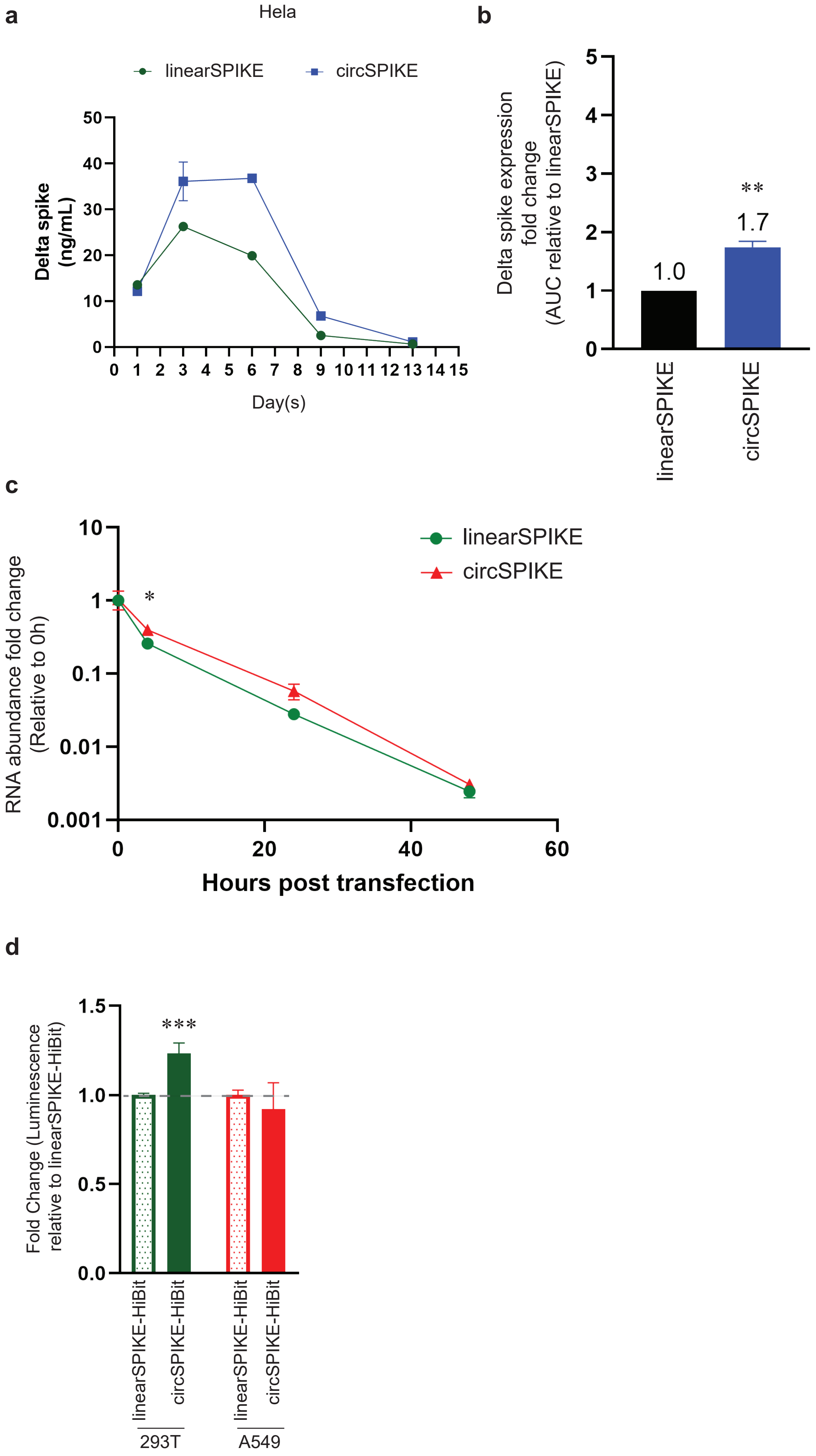

Figure S4. Comparison between linearSPIKE and circSPIKE.

(A) Hela cells were transfected with linearSPIKE and circSPIKE (circularized at 55°C – 15mM $MgCl_2$, 50mM Tris-HCl pH 7.0 and 1mM DTT) and supernatants were collected at indicated time points for ELISA assay to determine protein expression. (B) Quantification of protein expression from linearSPIKE and circSPIKE transfected Hela cells by measuring area under the curve (AUC). Data are presented as fold change relative to linearSPIKE. (C) Capped linearSPIKE RNA with 100% N1-methylpseudouridine or circSPIKE RNA was transfected into Hela cells. Transfected cells were harvested at indicated time points. Total RNA was isolated and spike transcript abundance was measured by RT-qPCR. Data are presented relative to 0h post-transfection. Statistical significance was determined using a two-tailed t test: ∗, $p < 0.05$. (D) LinearSPIKE-HiBit RNA that has a cap and 100% N1-methylpseudouridine and circSPIKE-HiBit RNA were transfected into 293T and A549 cells. A HiBit tag was included at the C-terminus for testing protein expression by luminescence measurement. Approximately 3 days post transfection, supernatants were harvested for luminescence measurement. Data are presented as fold change relative to linearSPIKE-HiBit. Statistical significance was determined using a two-tailed t test: ∗∗, $p < 0.01$; ∗∗∗, $p < 0.001$.

Supplementary Figure 5

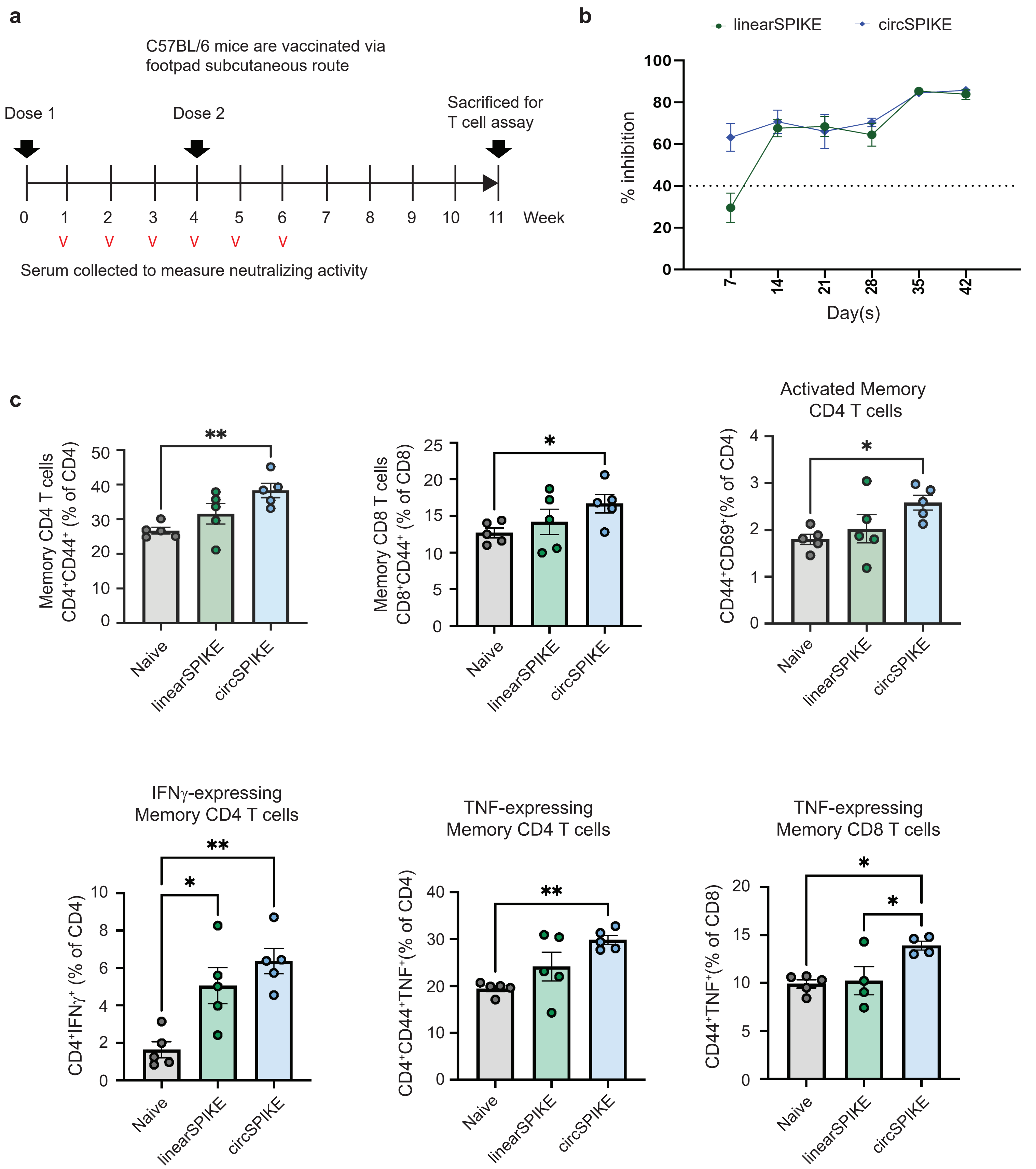

Figure S5. Circular spike RNA is a promising vaccine candidate against SARS-CoV2.

(A) Vaccination scheme and experimental design. (B) linearSPIKE RNA that has a cap and 100% N1-methylpseudouridine and circSPIKE RNA (circularized at 55°C – 15mM $MgCl_2$, 50mM Tris-HCl pH 7.0 and 1mM DTT) were formulated with LNP and were injected into C57BL/6 mice via footpad subcutaneously on day 1 and 28. Serum samples were collected at indicated time points to measure neutralizing activity. (C) Spleens of vaccinated mice were harvested 11 week after the administration of 1st dose and T cell response was characterized using various markers. Each dot represents one mouse. Statistical significance was determined using ANOVA: ∗ $p<0.05$; ∗∗ $p<0.01$.

Supplementary Figure 6

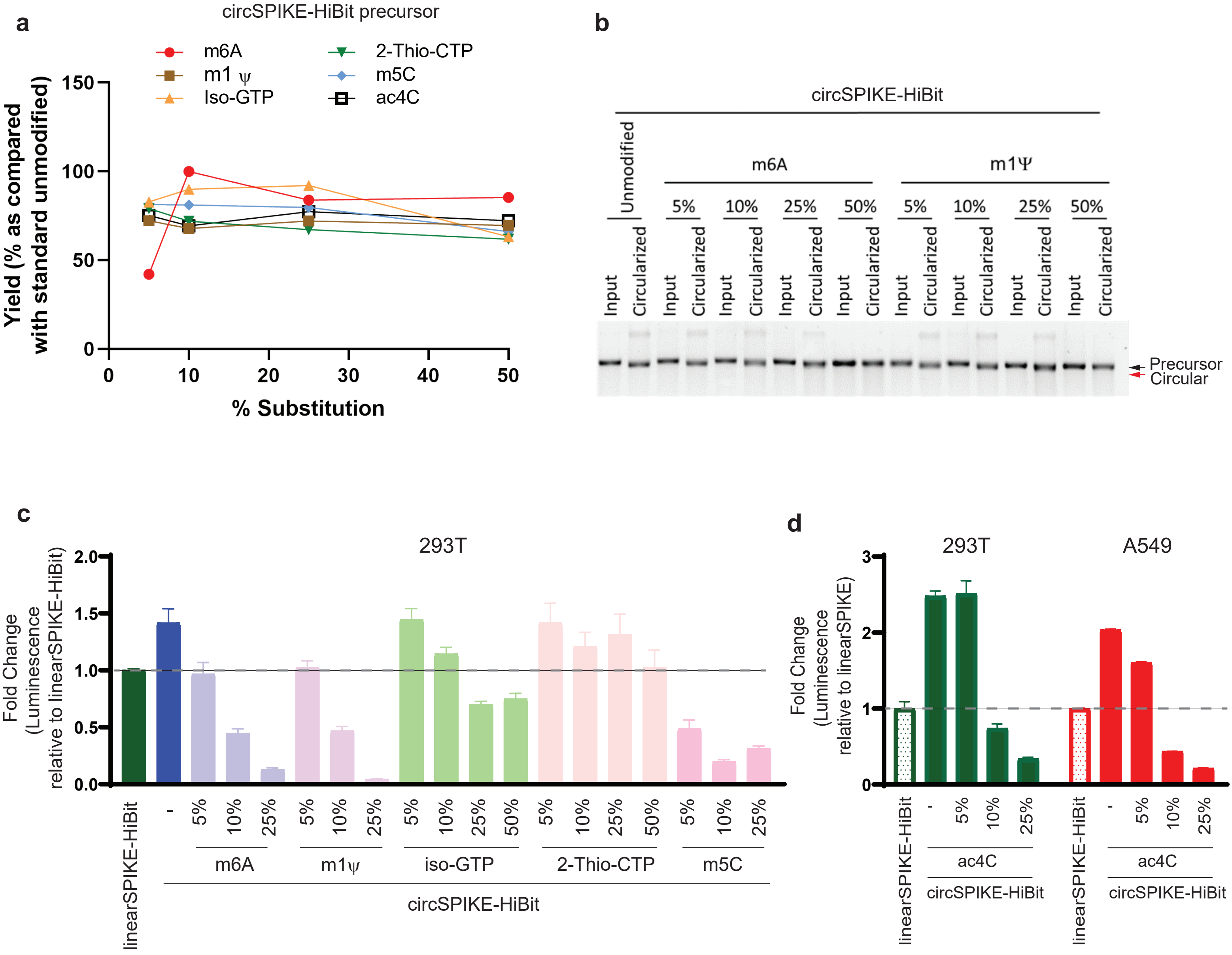

Figure S6. Effects of modified nucleotides on IVT yield, circularization and protein expression.

(A)(B) IVT yield and circularization efficiency of indicated RNA with various percentages of modified nucleotides. (C) RNA was transfected into 293T cells. Approximately 72h post transfection, supernatants were collected for luminescence measurement to determine protein expression. (D) Indicated RNA was transfected into 293T and A549 cells. About 3 days after transfection, supernatants were harvested for luminescence measurement to determine protein expression. Data are presented as fold change relative to linearSPIKE-HiBit and error bars indicate S.E.M.

Supplementary Figure 7

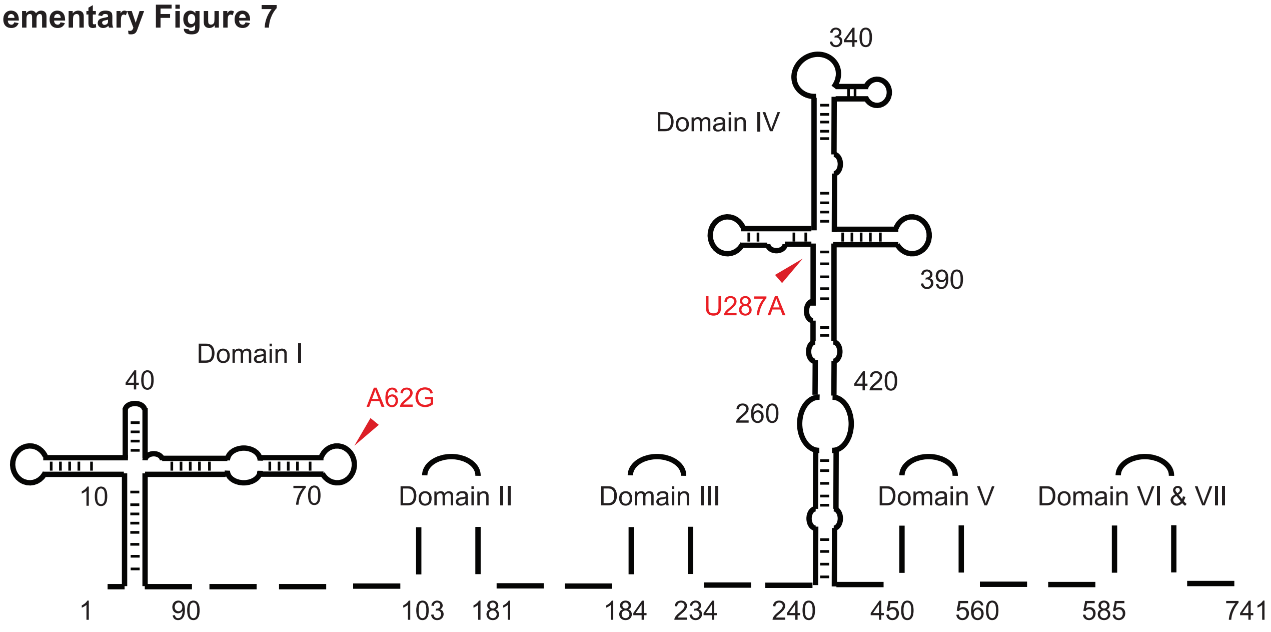

Figure S7 Secondary structure diagram of CVB3 IRES.

Functional mutations are indicated by red arrows. A62G is located in Domain I and U287A is located in Domain IV. Other domains are omitted for clarity and numbers indicate the nucleotide position in CVB3 IRES.

Supplementary Figure 8

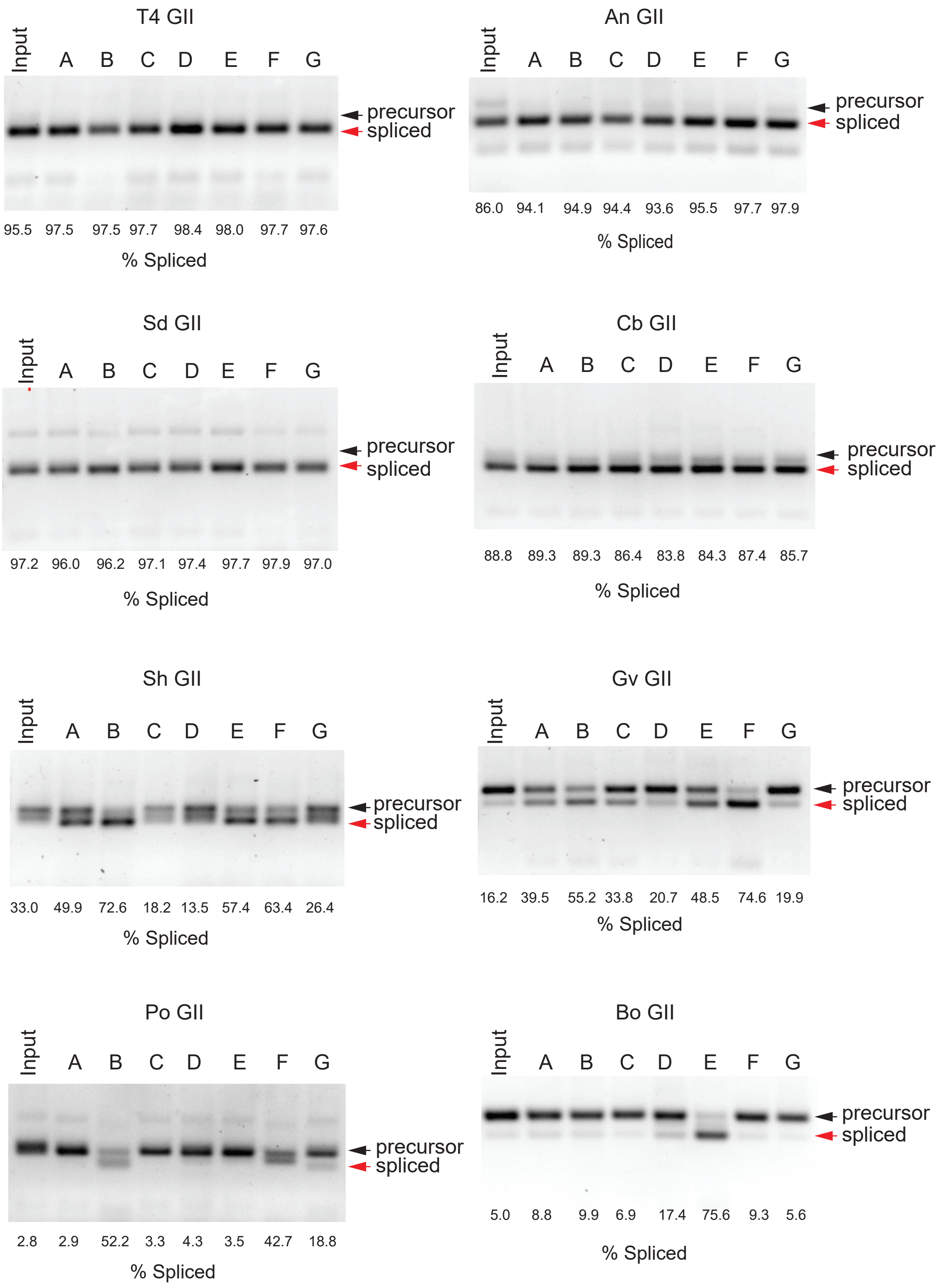

Figure S8. Characterization of group I intron self-splicing in the native form (GII).

T4, An, Sd, Cb, Sh, Gv, Po, Bo group I intron (GII) RNAs were in-vitro transcribed and folded under various conditions. All these samples were column purified and were analyzed by agarose gel. Black arrow indicates precursor and red arrow indicates spliced products. Densitometry analysis was performed to determine splicing efficiency.

Supplementary Figure 9

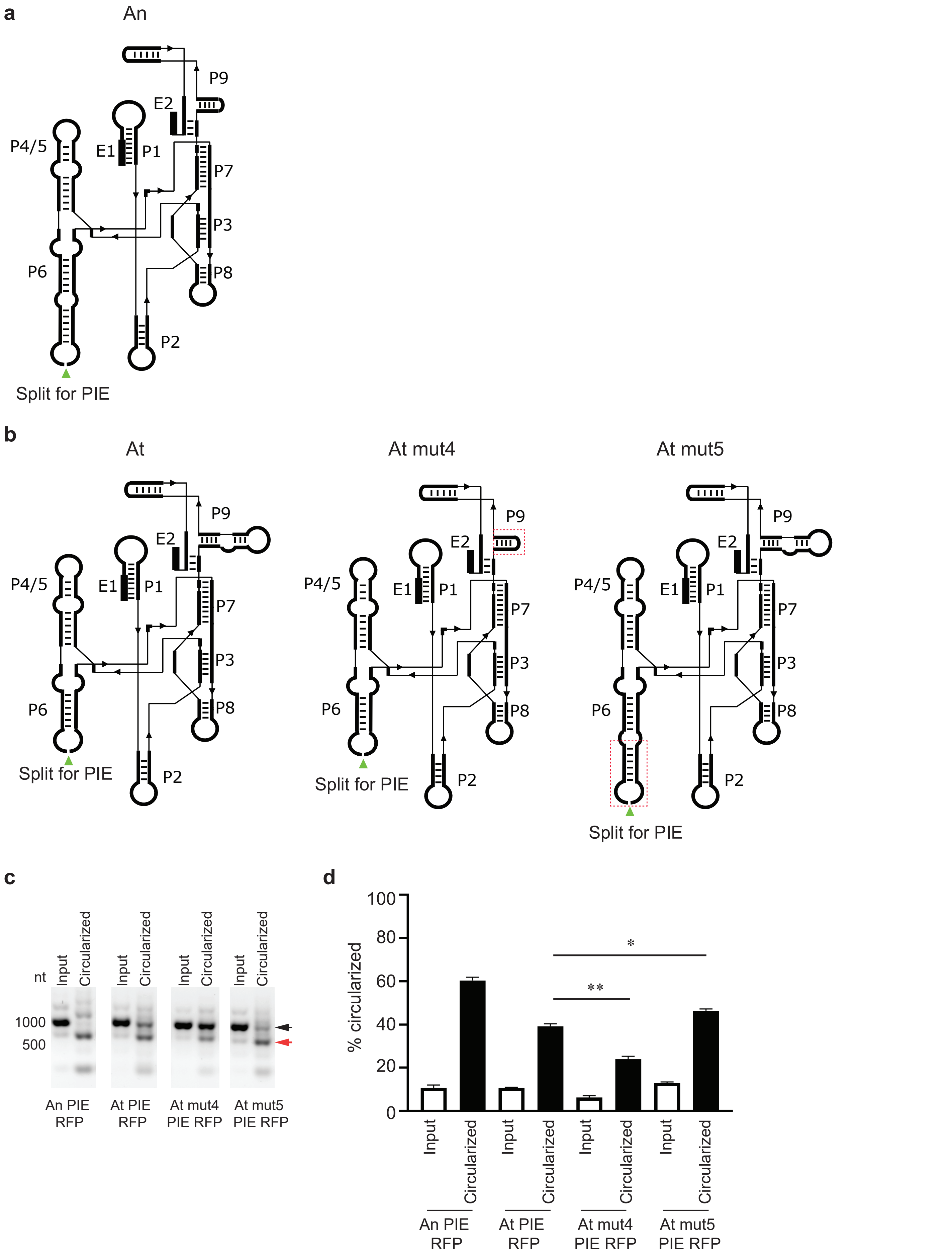

Figure S9. Mutational analysis of group I intron sequences in PIE circularization.

(A)(B) Schematics of An and At group I introns. (C) Indicated RNA was in-vitro transcribed and circularized at 55°C – 25mM NaCl, 15mM $MgCl_2$, 25mM HEPES pH7.5. Resultant samples were purified by columns and were analyzed by agarose gel electrophoresis. Black arrow indicates RNA precursor and red arrow indicates circularized RNA. (D) Densitometry analysis was performed to quantify the circular RNA band intensity. Data are mean ± S.E.M from two or three independent experiments, and representative images are shown. Statistical significance was determined using a two-tailed t test: $*$, $p < 0.01$; $**$, $p < 0.01$. At mut4 contains a shortened P9 domain as compared to At and At mut5 has an extended P6 domain.

Supplementary Figure 10

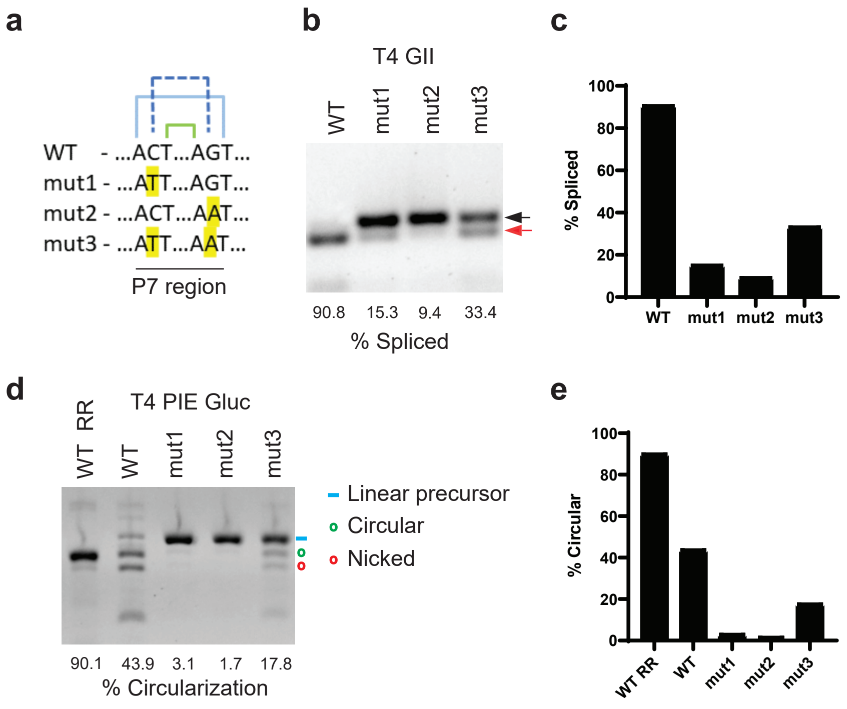

Figure S10. Mutational analysis of group I intron self-splicing in the native form (GII) and circularization in the permuted intron-exon system (PIE).

(A)Mutations made in the P7 region. (B)(C)(D)(E) Indicated RNAs were in-vitro transcribed and folded at 55°C – 25mM NaCl, 15mM $MgCl_2$, 25mM HEPES pH7.5. Resultant RNA samples were purified by columns and were analyzed by agarose gel electrophoresis or E-gel EX electrophoresis. Black arrow indicates precursor and red arrow indicates spliced products. Densitometry analysis was performed to quantify indicated band intensity using ImageJ.

Supplementary Figure 11

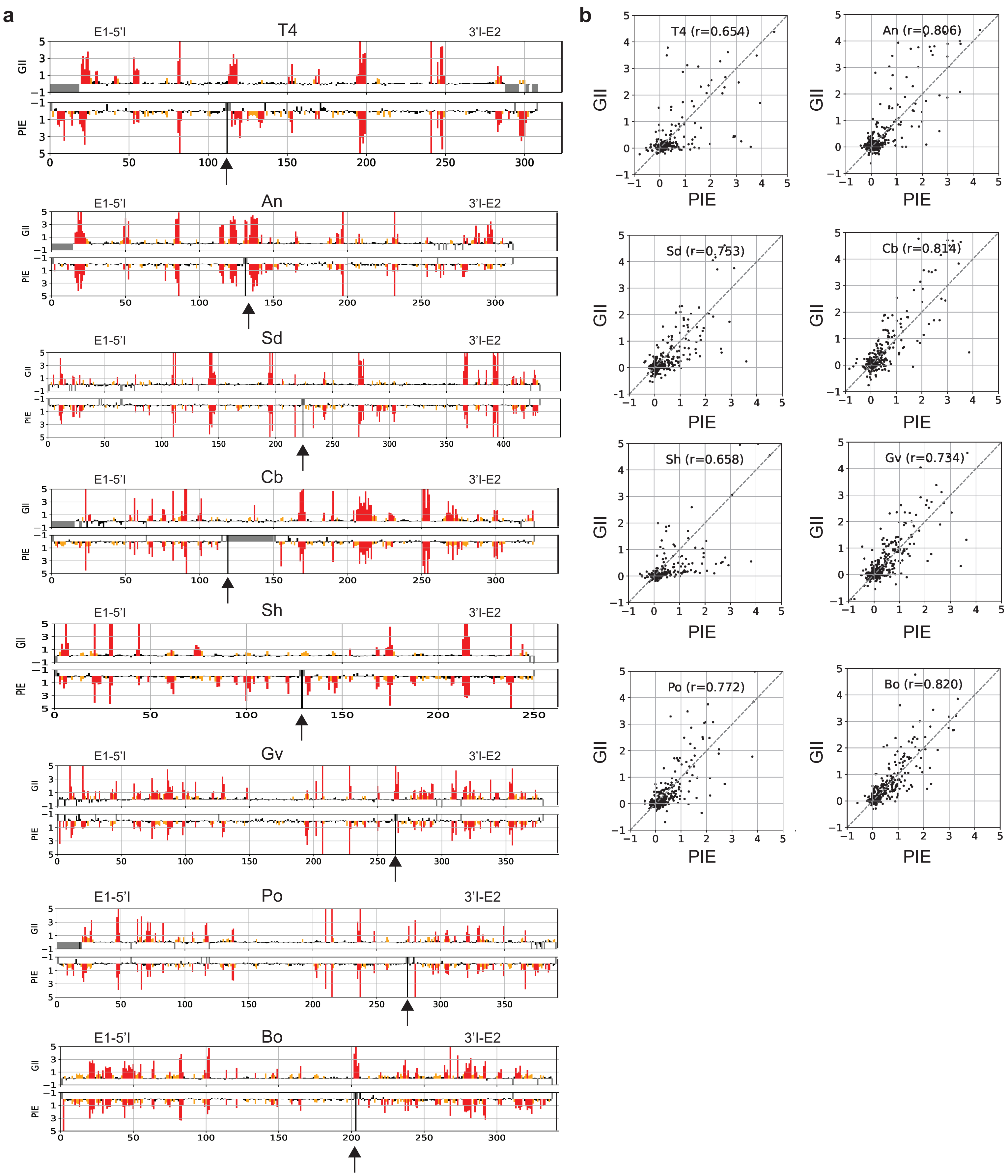

Figure S11. Structural mapping of various group I intron RNA in their native form (GII) and in the permuted intron-exon system (PIE).

(A) SHAPE-MaP reactivities of T4, An, Sd, Cb, Sh, Po, Gv, and Bo GII and PIE RNA. (B) Correlation analysis of SHAPE-MaP reactivities of 8 GII and PIE RNA.

Supplementary Figure 12

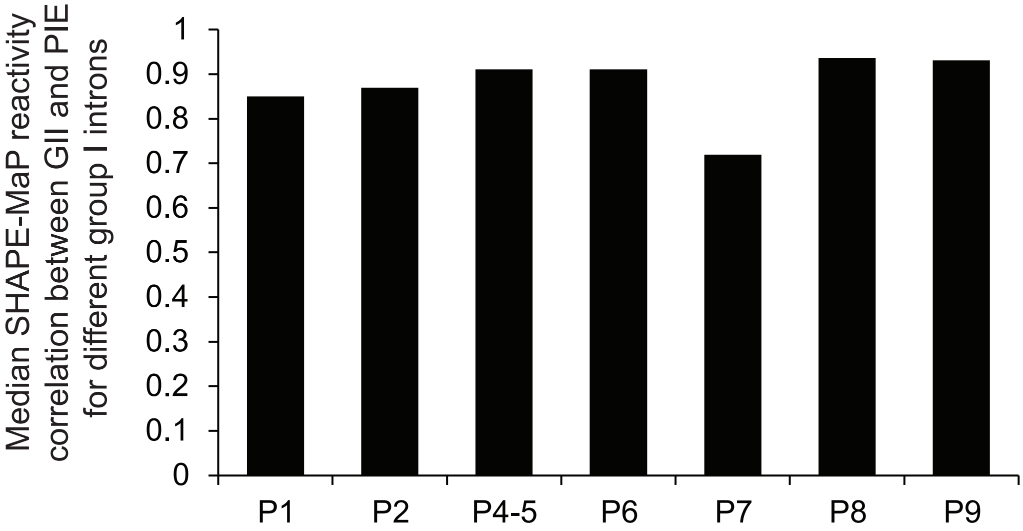

Figure S12. Median SHAPE-MaP reactivity correlation between GII and PIE for all 8 different group I introns by P1-P9 domains

Supplementary Figure 13

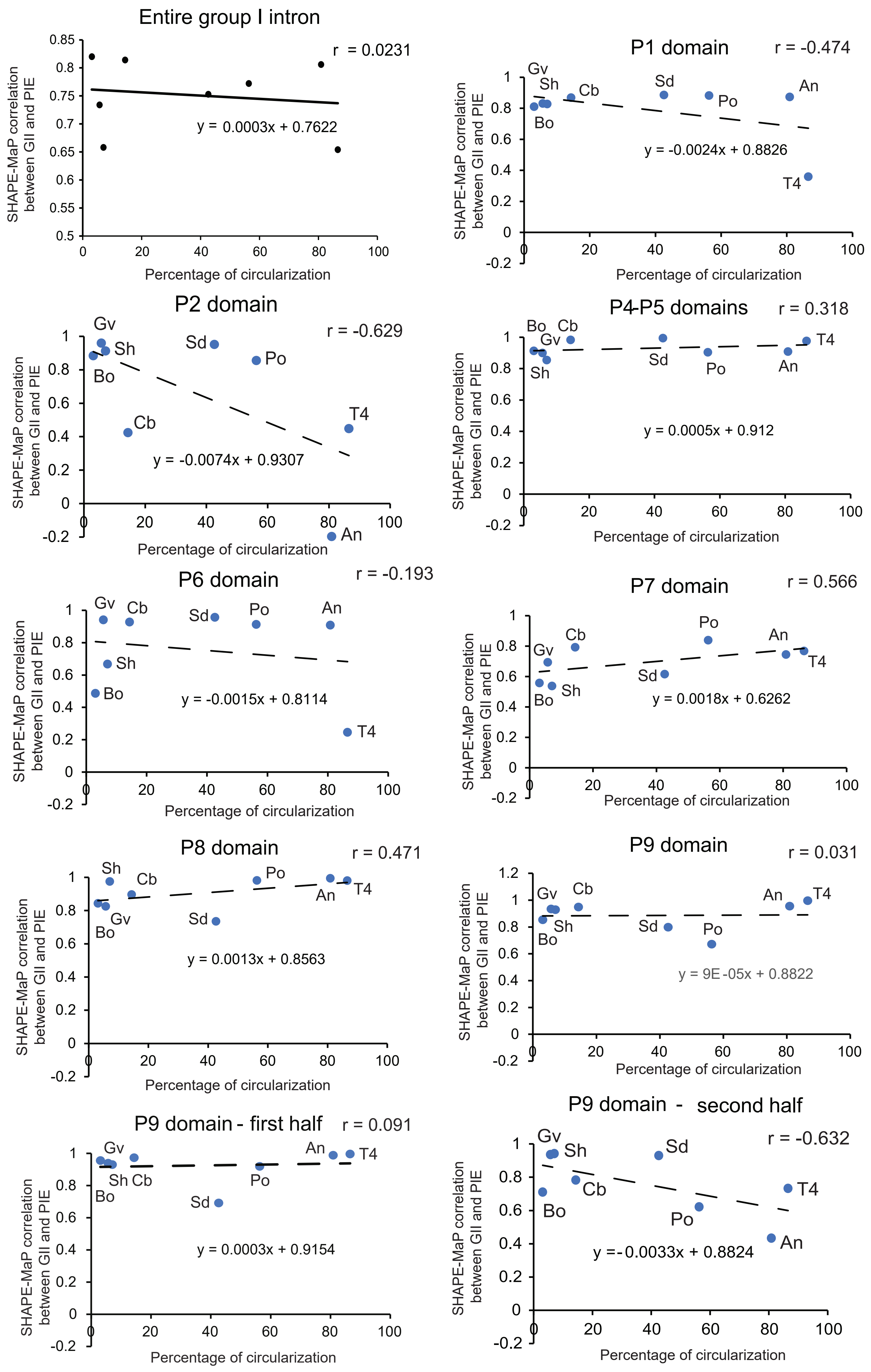

Figure S13. Correlations between circularization percentage and the SHAPE-MaP reactivity correlation between GII and PIE for the entire region and P1-P9 domains of T4, An, Sd, Cb, Sh, Gv, Po and Bo sequences.

This percentage of circularization is the circularization efficiency of T4, An, Sd, Cb, Sh, Gv, Po, and Bo PIE Gluc at 55°C in buffers containing 15mM $MgCl_2$, 50mM Tris-HCl pH 7.0 and 1mM DTT.

Supplementary Figure 14

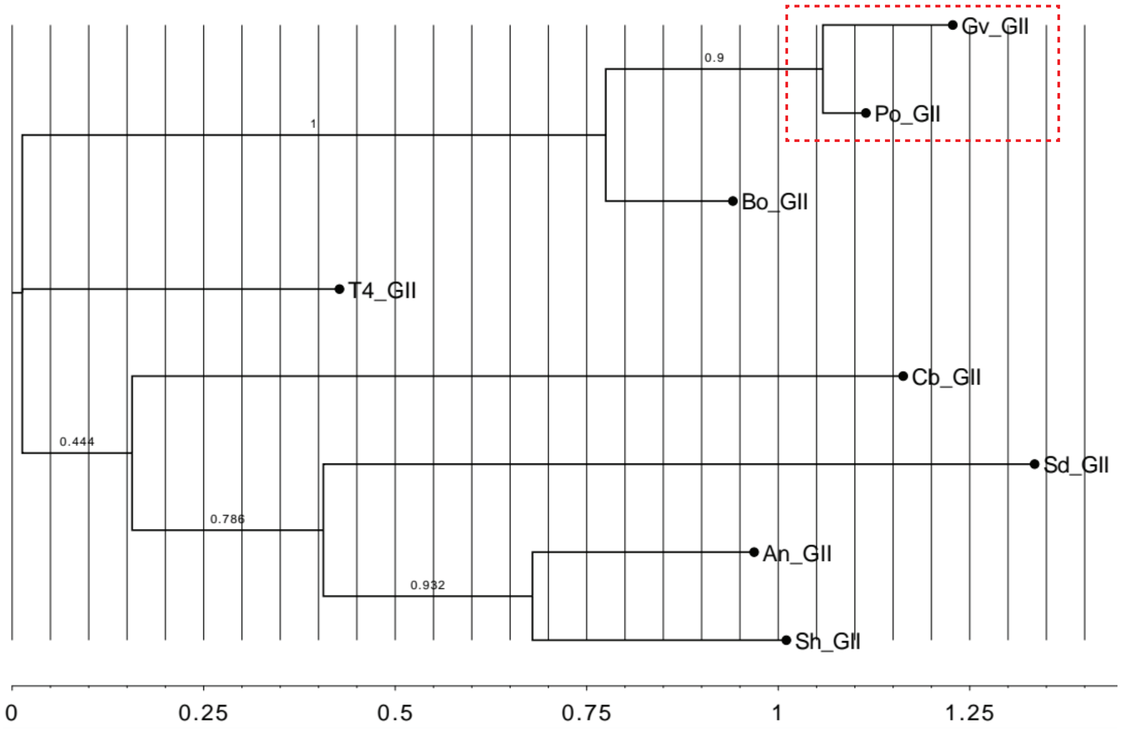

Figure S14. Phylogenetic analysis of 8 group I intron sequences.

Supplementary Figure 15

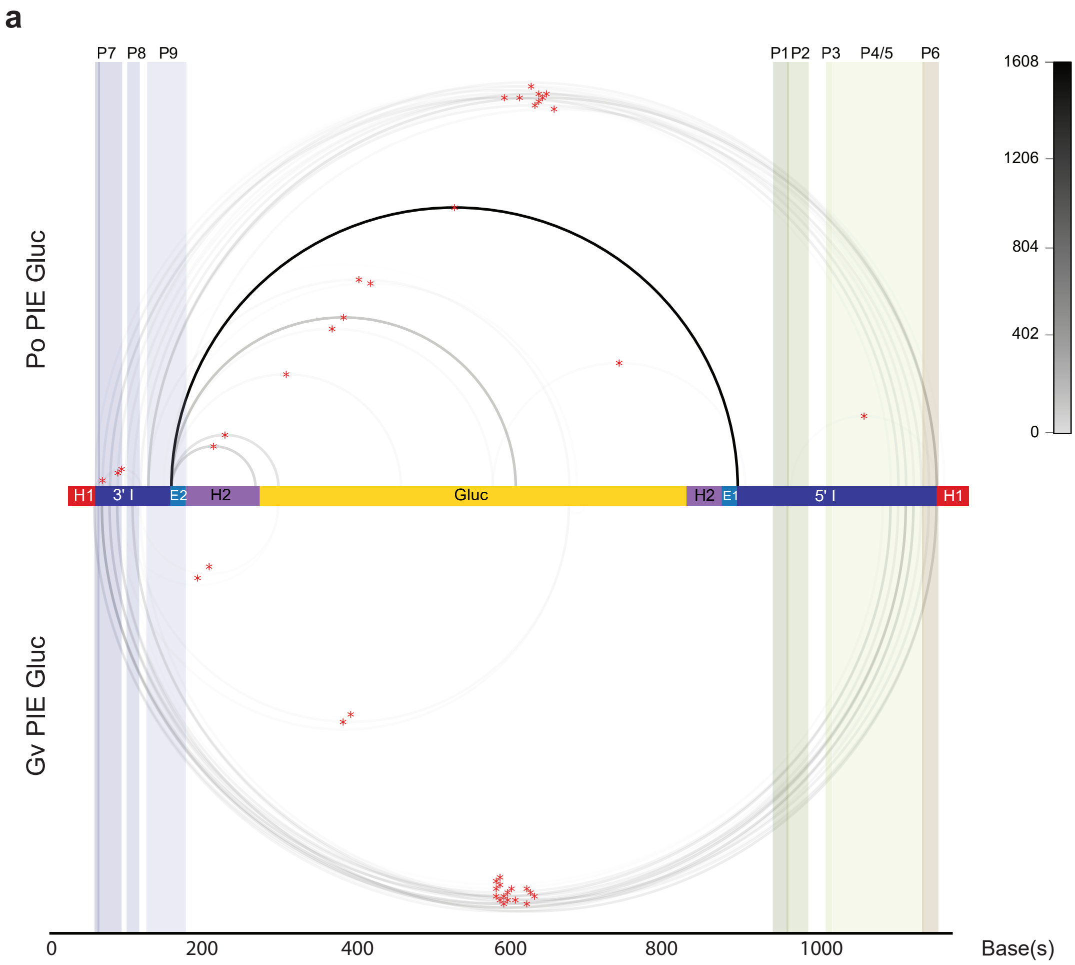

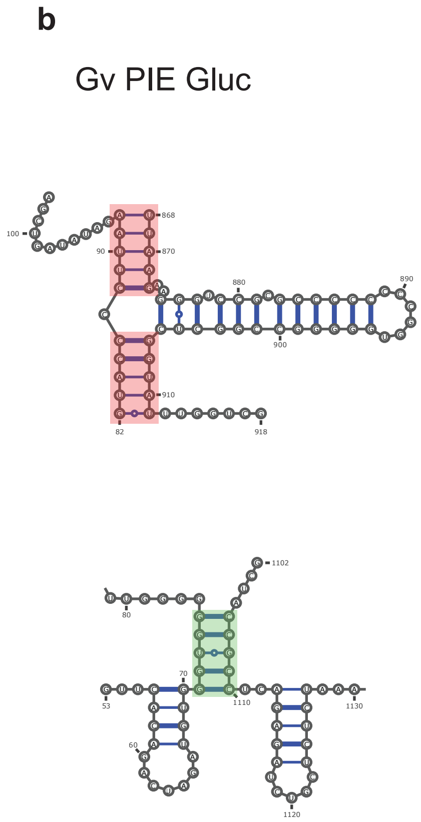

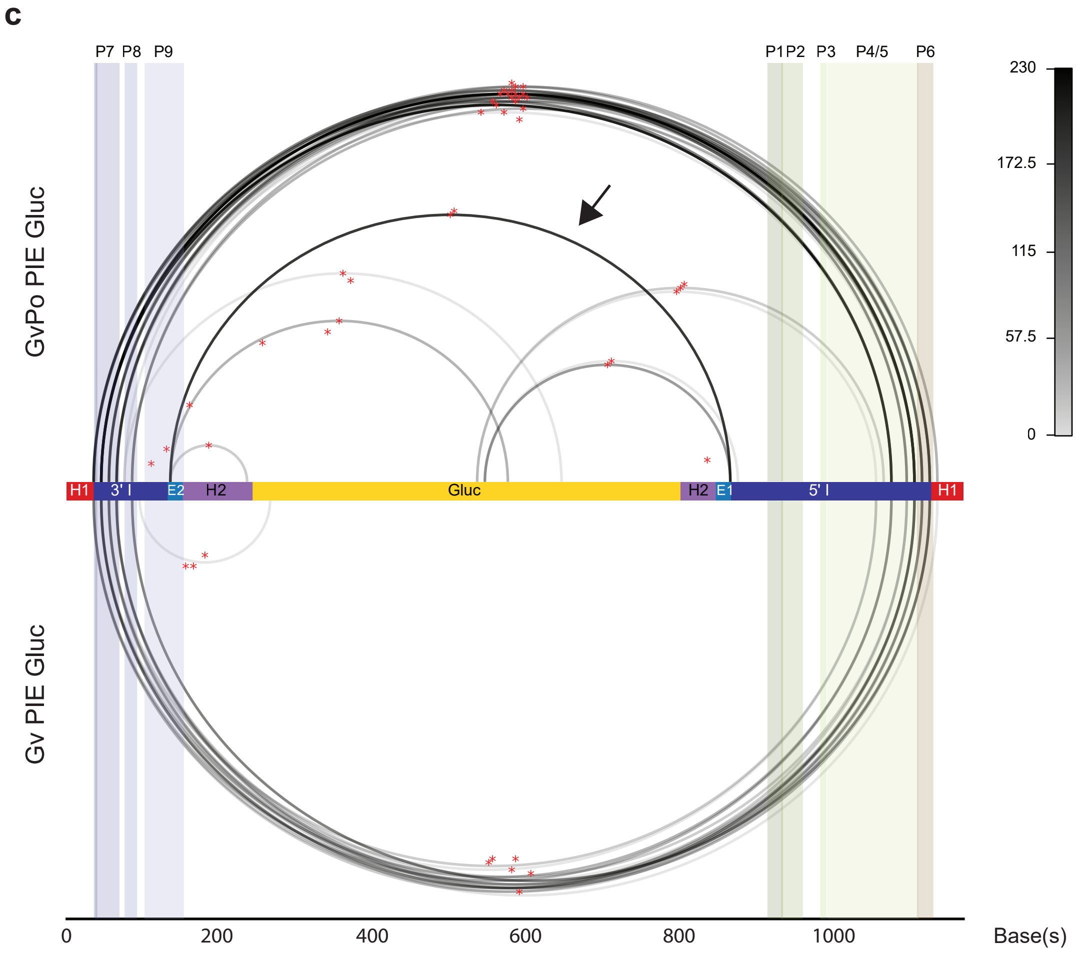

Figure S15. Mapping of molecular interactions.

(A) Differential analysis between Po PIE Gluc and Gv PIE Gluc was performed using Student's t-test and statistical significance (p value < 0.05) was indicated with red asterisks in arc plots. (B) Base-pairing regions of Gv PIE Gluc from RNAcofold analysis are highlighted in red and green. (C) Differential analysis between GvPo PIE Gluc and Gv PIE Gluc and statistical significance (p value < 0.05) was indicated with red asterisks in arc plots using Student's t-test. Colour bar in (A) and (C) indicates the scale of normalized chimeric read count in each construct. The arrow indicates the restored base-pairing in GvPo PIE Gluc.

Supplementary Figure 16

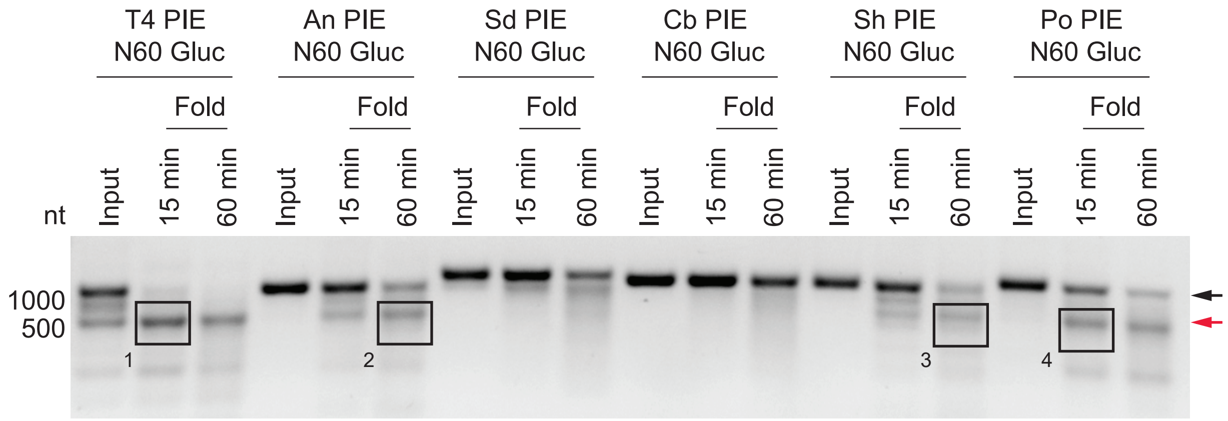

Figure S16. Determining E1 and E2 sequence requirement for PIE circularization.

Indicated IVT RNA was folded in a buffer containing 25mM NaCl, 15mM $MgCl_2$, 25mM HEPES pH7.5 at 55°C and was column purified. Resulting samples were analyzed by agarose gel electrophoresis. Indicated areas were gel extracted for subsequent processing and deep sequencing. Black arrow indicates linear precursor and red arrow indicates circularized RNA.

Supplementary Figure 17

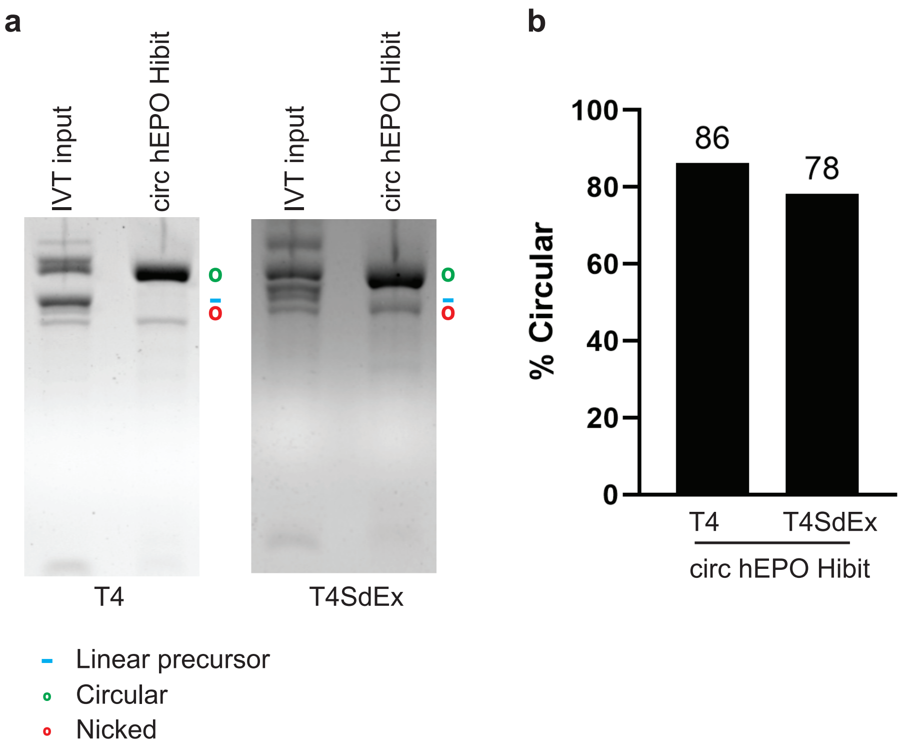

Figure S17. Generation of T4 and T4SdEx circ hEPO Hibit RNA.

(A) Indicated circular RNA was generated (circularized at 55°C – 15mM $MgCl_2$, 50mM Tris-HCl pH 7.0 and 1mM DTT) and was analyzed by E-gel EX electrophoresis. (B) Densitometry analysis was performed to quantify the circular RNA band intensity.

SUPPLEMENTARY TABLES

Supplementary Table 1: Construct and primer sequences

Supplementary Table 2: IRES engineering results

Supplementary Table 3: SHAPE-MaP reactivity of different group I intron sequences in native GII or in PIE system

Supplementary Table 4: SPLASH (pairwise interactions) normalized chimeric read count for Gv, Po and GvPo

SUPPLEMENTARY MATERIALS AND METHODS

Animal study and sera analysis post-vaccination

For mouse vaccination in C57BL/6 mice, groups of n=5 female mice were injected subcutaneously via footpad with 100ng LNP-RNA, and 4 weeks later, a second dose was administrated. The sera of immunized mice were collected every week and C57BL/6 mice were housed in the Duke-NUS vivarium and experiments were performed according to protocol approved by the SingHealth Institutional Animal Care and Use Committee. Neutralizing activities from these samples were determined using a modified version of the ImTracker-Multi COVID-19 viral variant neutralisation test (https://genybiologics.com/imtracker/)(1-3). This assay measures percentage inhibition of the ACE2-RBD interaction and is also used to qualitatively assess for the presence of neutralising antibodies against the Delta receptor binding domain (RBD) as per manufacturer's protocol. In brief, Delta RBD was coated onto 96-well flat bottom MaxiSorp immunoplates (SPL Life Sciences #32296) for 30 min at room temperature. The immunoplates were then incubated for 1 hour with blocking buffer after being washed thrice with washing buffer. The serum samples were diluted 10 times in blocking buffer. The negative and positive controls were prepared as described in the protocol. Post-incubation period, the washing step was repeated. Based on the assay layout, the respective serum samples, positive and negative controls were added on the immunoplates for 45 minutes at room temperature. Another round of washing procedure was repeated after the primary incubation. Following this, the detector antibody was prepared and added as per protocol for 45 minutes incubation at room temperature, protected from light. Post-secondary incubation, washing step was repeated and TMB substrate was then added to the immunoplates. 1 M H_2SO_4 was added to stop the reaction after 3 minutes. and the optical density at 450 nm (OD_{450}) was then recorded using a microplate reader (Tecan 100M). The formula below was used to calculate the percentage inhibition values.

$$\text{Inhibition (\%)} = \frac{Readout\ (negative\ control) - Readout\ (sample)}{Readout\ (negative\ control)} \times 100$$

T cell proliferation assay

Spleens from C57BL/6 mice were collected 11 weeks after their initial vaccination. The T cell proliferation was adapted from (4,5) with some modifications. Shortly, single-cell suspensions made using a 70-µm cell strainer and red blood cells (RBC) were lysed using the RBC lysis buffer (Biolegend, 420302). Antigen presenting cells (JAWSII cells, ATCC, CRL-3612) were

prepared before the experiment at 5 × 10^4 cells per well in α-minimum essential medium, with 20% FBS, 1% penicillin and streptomycin, and granulocyte-macrophage colony-stimulating factor (GM-CSF) (5 ng/mL; Sigma). Purified spike protein was prepared according to a published protocol (6). Antigens (5 µg/well) were preincubated with JAWSII cells for 48 hours and then treated with mitomycin C (25 µg/mL) for 25 min, followed by washing to remove excess mitomycin C. Splenocytes (100 µL of 5 × 10^6 cells/mL) were added to each well without pooling of cells so that each replicate represented the results from an individual mouse. After 72 hours, cells were harvested and prepared for flow cytometry analysis. The following antibody panel was used: LIVE/DEAD™ Fixable Aqua Dead Cell Stain Kit (Thermofisher, L34957); for extracellular staining, anti-mouse CD3-PE (Biolegend, 100206), anti-mouse CD4-BV650 (BD Biosciences, 563232), anti-mouse CD8-AF700 (Invitrogen, MCD0829), anti-mouse CD69-FITC (BD Pharmingen, 553236), anti-mouse CD44-BV510 (BD Horizon, 563114); and for intracellular staining, anti-mouse IFNg-APC-Cy7 (Biolegend, 505850), anti-mouse Ki76-BUV395 (BD Horizon, 564071), goat anti-mouse TNFa-unconjugated (R&D Systems, AF-410-NA) with anti-goat AF647 (Abcam, Ab150131. The sample were acquired on the LSRFortessa cell analyzer (BD Biosciences) and the data were analysed with FlowJo software (version 10).

Analysis of circular RNA formation accuracy

Synthesis of first-stand cDNA from circular Gluc RNA was performed using SuperScript III reverse transcriptase (Thermo Fisher) with primer junction R according to manufacturer's instructions. Subsequently, this cDNA was used as template for PCR amplification of 6 junction fragments with respective T4, An, Sd, Cb, Sh, and Po junction F and R primers. Each junction PCR fragment was processed using NEBNext Ultra II DNA Library Prep Kit to construct libraries compatible for Illumina sequencing. Illumina Raw paired-end reads were trimmed and then mapped using BWA (7). The Python Pysam package (8) was used to calculate the mismatch, deletion and insertion rates from aligned BAM files. The events of mismatch, deletion and insertion on each read were calculated using custom scripts. The 10 nucleotides on each side of junction site were selected to evaluate the junction accuracy. To investigate sequences of full circular RNA, same cDNA template was used for full circular PCR amplification with respective T4, An, Sd, Cb, Sh and Po full circle F and R primers. Then these full circle fragments were sequenced using Nanopore ligation sequencing kit. For Nanopore data analysis, FAST5 files for each construct were base-called with Guppy version 6.0.6. Base-called sequences were aligned with Minimap2 (v2.24) (9) in unspliced mode (-x map-ont). The resulting aligned reads were filtered with samtools (v1.6) (10) to keep only primary alignments (-F 0x904) . Further filtered by samtools to keep only the full

length reads alignment at least including the 5’ starting 21th bases and 3’ ending 20th bases. The filtered BAM files were calculated for mismatch, deletion and insertion rates by custom scripts. Selected Gluc sequence was used as benchmark. All primer sequences are listed in the supplementary section.

REFERENCE

1. Lim, S.M., Cheng, H.L., Jia, H., Kongsuphol, P., B, D.O.S., Chen, M.W., Ng, S.Y., Gao, X., Turaga, S.P., Heussler, S.P. *et al.* (2022) Finger stick blood test to assess postvaccination SARS-CoV-2 neutralizing antibody response against variants. *Bioeng Transl Med*, **7**, e10293.
2. Kongsuphol, P., Jia, H., Cheng, H.L., Gu, Y., Shunmuganathan, B.D., Chen, M.W., Lim, S.M., Ng, S.Y., Tambyah, P.A., Nasir, H. *et al.* (2021) A rapid simple point-of-care assay for the detection of SARS-CoV-2 neutralizing antibodies. *Commun Med (Lond)*, **1**, 46.
3. Gu, Y., Shunmuganathan, B., Qian, X., Gupta, R., Tan, R.S.W., Kozma, M., Purushotorman, K., Murali, T.M., Tan, N.Y.J., Preiser, P.R. *et al.* (2023) Employment of a high throughput functional assay to define the critical factors that influence vaccine induced cross-variant neutralizing antibodies for SARS-CoV-2. *Sci Rep*, **13**, 21810.
4. O'Neill, A., Mantri, C.K., Tan, C.W., Saron, W.A.A., Nagaraj, S.K., Kala, M.P., Joy, C.M., Rathore, A.P.S., Tripathi, S., Wang, L.F. *et al.* (2024) Mucosal SARS-CoV-2 vaccination of rodents elicits superior systemic T central memory function and cross-neutralising antibodies against variants of concern. *EBioMedicine*, **99**, 104924.
5. Saron, W.A.A., Rathore, A.P.S., Ting, L., Ooi, E.E., Low, J., Abraham, S.N. and St John, A.L. (2018) Flavivirus serocomplex cross-reactive immunity is protective by activating heterologous memory CD4 T cells. *Science advances*, **4**, eaar4297.
6. Stadlbauer, D., Amanat, F., Chromikova, V., Jiang, K., Strohmeier, S., Arunkumar, G.A., Tan, J., Bhavsar, D., Capuano, C., Kirkpatrick, E. *et al.* (2020) SARS-CoV-2 Seroconversion in Humans: A Detailed Protocol for a Serological Assay, Antigen Production, and Test Setup. *Current protocols in microbiology*, **57**, e100.
7. Li, H. and Durbin, R. (2010) Fast and accurate long-read alignment with Burrows-Wheeler transform. *Bioinformatics*, **26**, 589-595.
8. Heger A, B.T., Finkernagel F, Goodstadt L, Goodson M, Jacobs KB, Lunter G, Martin M, Schiller B. . (2016) Pysam: Python Interface for the SAM/BAM Sequence Alignment and Mapping Format. via PyPI.
9. Li, H. (2018) Minimap2: pairwise alignment for nucleotide sequences. *Bioinformatics*, **34**, 3094-3100.
10. Li, H., Handsaker, B., Wysoker, A., Fennell, T., Ruan, J., Homer, N., Marth, G., Abecasis, G., Durbin, R. and Genome Project Data Processing, S. (2009) The Sequence Alignment/Map format and SAMtools. *Bioinformatics*, **25**, 2078-2079.
